# Stochastic Loss of Silencing of the Imprinted *Ndn/NDN* Allele, in a Mouse Model and Humans with Prader-Willi Syndrome, Has Functional Consequences

**DOI:** 10.1371/journal.pgen.1003752

**Published:** 2013-09-05

**Authors:** Anne Rieusset, Fabienne Schaller, Unga Unmehopa, Valery Matarazzo, Françoise Watrin, Matthias Linke, Beatrice Georges, Jocelyn Bischof, Femke Dijkstra, Monique Bloemsma, Severine Corby, François J. Michel, Rachel Wevrick, Ulrich Zechner, Dick Swaab, Keith Dudley, Laurent Bezin, Françoise Muscatelli

**Affiliations:** 1 INSERM, Institut de Neurobiologie de la Méditerranée (INMED) U901, Marseille, France; 2 Aix-Marseille Université, INMED UMR901, Marseille, France; 3 Netherlands Institute for Neuroscience, an Institute of the Royal Netherlands Academy of Arts and Sciences, Amsterdam, The Netherlands; 4 Universitätsmedizin der Johannes Gutenberg-Universität Mainz Institut für Humangenetik, Mainz, Germany; 5 INSERM, U1028, CNRS, UMR5292, Université Claude Bernard Lyon 1, Lyon Neuroscience Center, Villeurbanne, France; 6 Department of Medical Genetics, University of Alberta, Edmonton, Alberta, Canada; Queensland Institute of Medical Research, Australia

## Abstract

Genomic imprinting is a process that causes genes to be expressed from one allele only according to parental origin, the other allele being silent. Diseases can arise when the normally active alleles are not expressed. In this context, low level of expression of the normally silent alleles has been considered as genetic noise although such expression has never been further studied. Prader-Willi Syndrome (PWS) is a neurodevelopmental disease involving imprinted genes, including *NDN*, which are only expressed from the paternally inherited allele, with the maternally inherited allele silent. We present the first in-depth study of the low expression of a normally silent imprinted allele, in pathological context. Using a variety of qualitative and quantitative approaches and comparing wild-type, heterozygous and homozygous mice deleted for *Ndn*, we show that, in absence of the paternal *Ndn* allele, the maternal *Ndn* allele is expressed at an extremely low level with a high degree of non-genetic heterogeneity. The level of this expression is sex-dependent and shows transgenerational epigenetic inheritance. In about 50% of mutant mice, this expression reduces birth lethality and severity of the breathing deficiency, correlated with a reduction in the loss of serotonergic neurons. In wild-type brains, the maternal *Ndn* allele is never expressed. However, using several mouse models, we reveal a competition between non-imprinted *Ndn* promoters which results in monoallelic (paternal or maternal) *Ndn* expression, suggesting that *Ndn* allelic exclusion occurs in the absence of imprinting regulation. Importantly, specific expression of the maternal *NDN* allele is also detected in post-mortem brain samples of PWS individuals. Our data reveal an unexpected epigenetic flexibility of PWS imprinted genes that could be exploited to reactivate the functional but dormant maternal alleles in PWS. Overall our results reveal high non-genetic heterogeneity between genetically identical individuals that might underlie the variability of the phenotype.

## Author Summary

Genomic imprinting is a process that causes genes to be expressed from only one of the two chromosomes, according to parental origin, the other copy of genes being silent. Prader-Willi Syndrome (PWS) is a neurodevelopmental disease involving imprinted genes, including *NDN*, which are only expressed from the paternally inherited chromosome, the maternally inherited copy of the gene normally being silent. Here we show that, in absence of the paternal *Ndn* copy only, the maternal *Ndn* allele is expressed at an extremely low level with a high degree of heterogeneity. The level of this expression is dependent on both the sex of the offspring and the genotype of the mother. In about 50% of mutant mice, this expression reduces birth mortality and severity of the breathing deficiency, showing a functional role of this low expression. Importantly, specific expression of the maternal *NDN* allele is also detected in post-mortem brain samples of PWS individuals. Our data reveal an unexpected epigenetic flexibility of PWS imprinted genes that could be exploited to reactivate the functional but dormant maternal alleles in PWS. Overall our results reveal high non-genetic heterogeneity between genetically identical individuals that might contribute to variability in the phenotype.

## Introduction

Imprinted genes are functionally mono-allelic in a parent-of-origin specific manner. Genomic imprinting is a non-Mendelian epigenetic form of gene regulation which is germline-inherited since the epigenetic marks are established in the parental gametes without altering the DNA sequence [1]. Compared to most other tissues the brain is enriched in genes showing an imprinted pattern of expression [2], vulnerable to environmental perturbation [3] and contributing to various neurodevelopmental diseases [4], [5]. This vulnerability, linked to a plasticity of gene regulation, might also allow a positive adaptation of an organism to a new external environment. It is important to examine situations in which partial loss of imprinting (LOI) rescues a mutant phenotype. Understanding the mechanisms underlying this positive effect could lead to therapeutic avenues that manipulate this rheostat function.

*Necdin* (*Ndn*) is an imprinted gene present in both human and mouse, and its maternally inherited allele is normally silenced [6]–[9]. The human *NDN* gene is located in a large imprinted domain. All the paternally expressed genes from this domain are candidate genes for some of the symptoms of Prader-Willi Syndrome (PWS), an orphan neurodevelopmental genetic disease [10] (OMIM 176270). The essential clinical diagnostic criteria include neonatal hypotonia and abnormal feeding behavior with a poor suck followed by a hyperphagia, resulting in severe obesity, and behavioral problems [11]–[14]. Breathing deficiency is a significant health concern for many patients and contributes to some cases of sudden death [15], [16]. Notably, there is considerable variability in symptom severity among patients [11].

Mouse strains with targeted inactivation of single PWS genes have been created, and heterozygous mice with a paternally inherited deficiency (+m/−p) are generally considered to be functionally null. Four independent *Ndn*-deficient mouse lines have been created [9], [17]–[19], three of which display PWS associated phenotypes [9], [17]–[21] including partial early post-natal lethality due to respiratory distress [20], [22].

In Muscatelli's *Ndn*-KO mouse model (named Ndn^tm1.1Mus^), we observed a high level of phenotypic heterogeneity among the *Ndn+m/−p* mice within each litter; notably in the incidence and severity of apneas [22]. The stochastic nature of gene expression can generate pronounced phenotypic variations [23] and here we hypothesize that this inter-individual variability, among *Ndn+m/−p* mice, might result from a “stochastic” activation of the putatively silent maternal allele of *Ndn*.

In this study, we investigate this hypothesis by comparing homozygous mice deleted for both alleles of *Ndn* (*Ndn*−/−) with heterozygous *Ndn* (*Ndn+m/−p*) mice. We perform a comprehensive analysis of the *in vivo* expression and functional role of the *Ndn* maternal allele. We investigate the genetic context and the mechanism underlying this maternal expression. Finally, we show that the maternal allele of *NDN* is transcribed and that Necdin protein is present in human *post-mortem* Prader-Willi brains.

## Results

### *Ndn−/−* mutant mice present a more severe phenotype than *Ndn+m/−p* littermates

After 36 backcrosses on the C57Bl/6J genetic background, we measured the lethality of *Ndn+m/−p* mice versus *Ndn+/+* mice, both derived from crosses between a wild-type (WT) female and a heterozygous male deleted for the *Ndn* maternal allele (−m/+p). As expected [17], *Ndn+m/−p* mice were significantly under-represented at weaning (28% reduction, 125 +/+ versus 91 *Ndn+m/−p*; CHI^2^ test, P<0.01). Furthermore, we confirmed that there was an equivalent number of *Ndn+m/−p* (118) versus *Ndn+/+* (116) pups at birth, and 21% lethality between postnatal day (P) P0 and P3 in both sexes. However, in a cohort of 75 *Ndn*−/− mutants, derived from crosses between a *Ndn−/−* female and a *Ndn−/−* male, we found a 43% lethality of the *Ndn−/−* pups (32/75), between P1 and P2, and these pups were visibly cyanotic. Altogether, these data suggest that, due presumably to respiratory deficiency, *Ndn−/−* newborns are twice as likely to die early compared to *Ndn+m/−p* newborns. This result is surprising because in theory there is no *Ndn* expression in either *Ndn*−/− or *Ndn+m/−p* pups.

Next, we compared breathing pattern between *Ndn*−/− and *Ndn+m/−p* mice. Previously, we demonstrated that newborn and young adult *Ndn+m/−p* mice present an irregular respiratory rhythm with frequent apneas [22]. Importantly, such apneas were more than twice as frequent in *Ndn−/−* compared to *Ndn+m/−p* mice (Table 1).

**Table 1:** Respiratory pattern and apnea in *Ndn*−/− young adult versus *Ndn*+m/−p mice.

	*Ndn+m/−p*	*Ndn*−/−
**Animal weight (g)**	16.7±2.3	16.4±2.7
**Minute Ventilation (ml.min^−1^.g^−1^)**	2.0±0.6	2.6±1.0
**Breathing Frequency (BPM)**	256.7±56.6	298.3±81.4
**Tidal Volume (ml.10^−3^.g^−1^)**	7.9±1.0	8.7±1.8
**Expired Volume (ml.10^−3^.g^−1^)**	7.8±1.0	8.7±1.8
**Expiratory Time (ms)**	174.9±28. 9	166.3±45.0
**Apnea number, per hour**	**8.8±8.9**	**18.2±14.4^a^**
**Apnea duration (ms)**	900±109	929±107
**% of animals with apnea >750 ms**	89%	84%
**Apnea scores (% time.hour^−1^)**	**0.2±0.2**	**0.5±0.4^b^**

While the mean apnea duration was similar between genotypes (∼900 msec), evaluation of the total apnea duration expressed as the total recording time (i.e. apnea scores) revealed a two-fold increase in the percentage of total apnea duration in *Ndn−/−* compared to *Ndn+m/−p* individuals.

Values are represented as Mean±SD; n = 20 for *Ndn+m/−p* and 18 for *Ndn* −/− mice.

Mann Whitney t-test, two-tailed. P value = 0.02 (a) and 0.03 (b).

In summary, the respiratory phenotype of *Ndn*−/− homozygous mice is more severe than in *Ndn+m/−p* heterozygotes, suggesting a role for the maternally inherited *Ndn* allele.

### Quantitative expression of the *Ndn* maternal allele

Since we suspected a role of the maternal *Ndn* allele in the phenotype of *Ndn+m/−p* mice, we further investigated the expression of this allele using a specific anti-Necdin antibody in immunoblot analyses of protein extracts from different P1 brains (Figure S1A) or from individual E12.5 embryos (Figure S1B). We detected a specific signal at the expected size for WT animals but also a fainter signal (10–20 fold less intense) in four of the eight *Ndn+m/−p* animals, with no signal in *Ndn−/−* mice.

Consistent with our previous study [17], we did not detect maternal *Ndn* allele expression by RT-PCR (Figure S1C). We therefore increased the experimental sensitivity using RT-qPCR. We focused on different developmental stages (E12.5, P1 and adult) [24], using total brain tissue as well as brain structures known to highly express *Ndn* (hypothalamus) or to be involved in respiratory function (pons). A total of 258 individual animals on a C57Bl/6J genetic background, including 72 WT, 57 *Ndn*−/− and 129 *Ndn+m/−p*, were analyzed.

**Figure 1:**
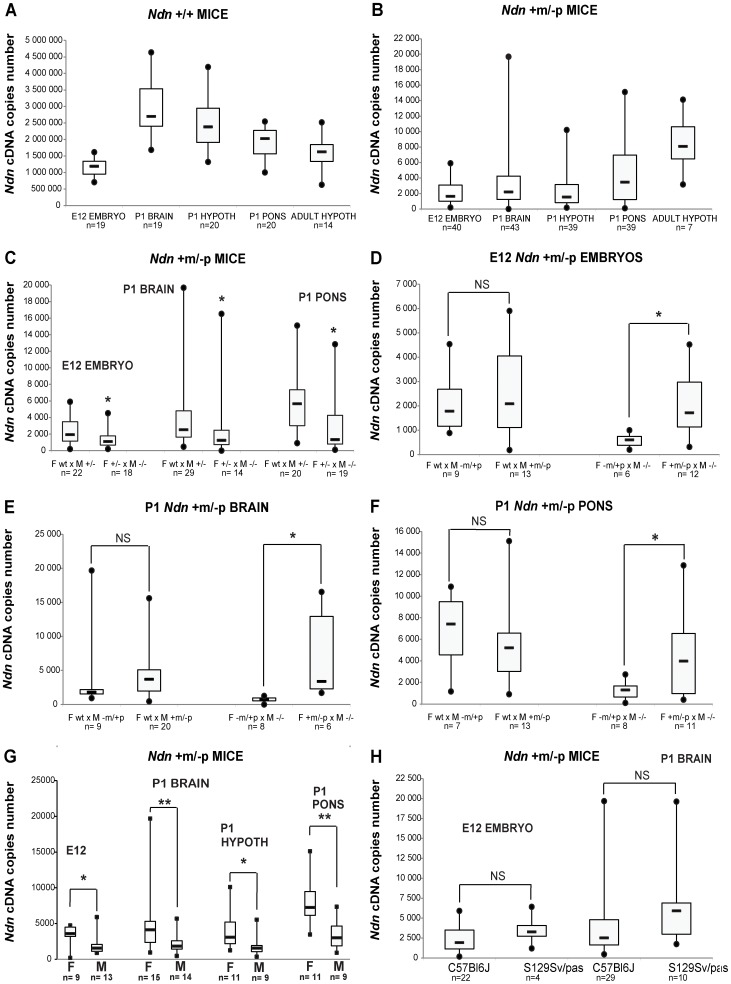
*Ndn* expression analyzed by RT-qPCR in *Ndn+m/−p* mice. RT-qPCR analysis of *Ndn* transcripts. A) *Ndn* transcripts in E12 WT embryos and selected brain tissues from P1 (whole brain, hypothalamus, pons) or adult (hypothalamus) WT mice. B) *Ndn* transcripts in *Ndn+m/−p* E12 embryos and brain tissues from P1 (whole brain, hypothalamus, pons) or adult (hypothalamus) *Ndn+m/−p* mice. C–F) Quantification of *Ndn* transcripts in *Ndn*+m/−p E12 embryos (C,D) and whole brain (C,E) and pons (C,F) from P1 mice with respect to parental genotype (C) and to the maternal or paternal genotype contribution indicating a grandparental influence (D–F). G) Quantification of *Ndn* transcripts in *Ndn+m/−p* E12 embryos, P1 and adult *Ndn+m/−p* brain tissues in male (M) and female (F) mice. H) Quantification of *Ndn* transcripts in *Ndn+m/−p* E12 embryos and *Ndn+m/−p* P1 mice in C57Bl/6J and S129Sv/Pas mouse strains. The *Ndn* transcript copy number in *Ndn+m/−p* offspring (E12, P1 brain, P1 pons; n = 71) issued from a cross between a WT female and a *Ndn*+/− male is significantly more than 2 fold higher than the *Ndn* copy number in *Ndn+m/−p* offspring (n = 51) issued from a *Ndn+/−* female crossed with a *Ndn−/−* male (C). Considering separately the effect of the maternal or paternal genotype, we showed that when the mother is WT and the father is (+/−), with a maternal *Ndn* mutant allele (−m/+p) or a paternal *Ndn* mutant allele (+m/−p), then there is no difference in the copy number of *Ndn* maternal transcripts between the *Ndn+m/−p* individuals of the offspring of both types of crosses (n = 25 and n = 46, respectively) (D,E,F). However, we observed an effect of the maternal genotype, with a significant difference in the level of *Ndn* maternal transcripts between the *Ndn+m/−p* individuals (n = 22) issued from a (−m/+p female X −/− male) compared with the *Ndn+m/−p* individuals (n = 29) issued from a (+m/−p female X −/− male) (D,E,F); the +/+ or *Ndn+m/−p* maternal genotype is correlated with a significant three times higher level of *Ndn* maternal expression in the *Ndn+m/−p* offspring. Values are represented as Median (Q1, Q3). WMW test, two-tailed. * P value<0.05 and ** <0.01.

In *Ndn−/−* mutant mice, no *Ndn* transcripts were detected irrespective of the brain structures or stages analyzed (data not shown). In WT individuals (Figure 1A), as expected [17], [24], we observed higher *Ndn* expression in P1 brains compared with expression in E12.5 embryos. In *Ndn+m/−p* individuals, maternal *Ndn* transcripts were detected, but the transcript level was reduced 800 (P1 brain) to 1500 (adult hypothalamus)-fold compared to WT individuals (comparing the medians, Figure 1B). Interestingly, there was a huge inter-individual variability (×100 to ×1000 between the extreme values) for all *Ndn+m/−p* mice, irrespective of the stages and tissues tested.

We searched for factors that influence the level of transcripts of the *Ndn* maternal allele. While our results show an absence of a significant effect of the paternal genotype (Figure 1D,E,F), the maternal genotype clearly influences maternal *Ndn* expression in the *Ndn+m/−p* offspring. Although in all cases the offspring (+m/−p) have inherited a wild-type (+m) allele from the mother, offspring from the *Ndn+/+* or *Ndn+m/−p* maternal genotype had a significant three-fold higher level of *Ndn* maternal expression compared to those from a *Ndn−m/+p* maternal genotype (Figure 1D,E,F). Importantly, the extensive variability of *Ndn* maternal expression is also positively correlated with both those maternal genotypes. In contrast, litters issued from *Ndn−*m/+p mothers showed both a lower level of *Ndn* expression and an absence of variability in the *Ndn+m/−p* offspring (Figure 1D,E,F). In addition, there is a gender-specific effect on the *Ndn* maternal expression in *Ndn+m/−p* offspring, with females expressing two-fold more *Ndn* expression compared to males (Figure 1G).

Finally we compared this maternal expression in *Ndn+m/−p* offspring from a C57Bl/6J or a 129Sv/Pas genetic background. In both mouse strains, a similar level of *Ndn* maternal expression was observed (Figure 1H).

We conclude that an extremely low but specific transcription of the maternally inherited *Ndn* allele in *Ndn+m/−p* individuals is found in at least two mouse strains (C57Bl/6J, 129Sv/Pas). Transcript numbers are highly variable irrespective of the developmental stage or the brain structure analyzed, even among littermates. Finally, the quantity of maternal *Ndn* transcripts depends significantly on the maternal genotype and on the gender.

### Qualitative expression of the *Ndn* maternal allele at different developmental stages

We asked whether maternal *Ndn* expression was due to: 1) low but homogeneous expression in all tissues and/or 2) reduced but focal expression in specific structures and cell types.

**Figure 2:**
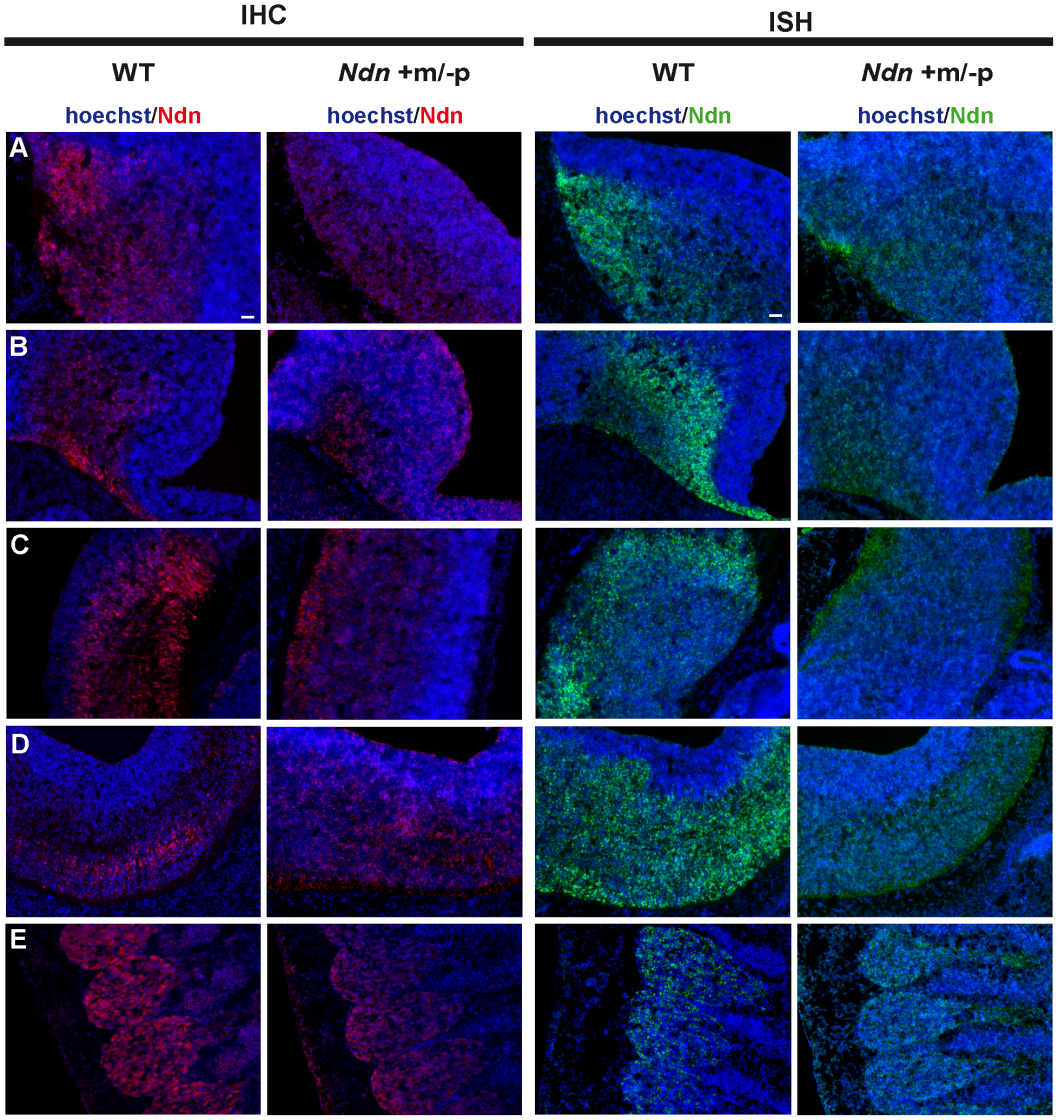
*Ndn* expression in *Ndn+m/−p* E12.5 embryos. Expression of *Ndn* in the nervous system of WT and *Ndn+m/−p* embryos at E12.5 revealed by IHC or ISH on frozen sections using a Necdin specific antibody (Ndn,red) or *Ndn* RNA probe (green). Tissue sections are visualized using a Hoechst labeling (blue). Expression is detected in both genotypes, at the protein and transcript levels, in the preoptic area (A), supraoptic area (B), thalamus (C), pons (D) and in the dorsal root ganglia (E). Note that the level of expression is weaker and more restricted in *Ndn+m/−p* embryos. Other structures, like the tegmentum, the subthalamus and the spinal cord also express the *Ndn* maternal allele. Scale bar: 50 µm.

**Figure 3:**
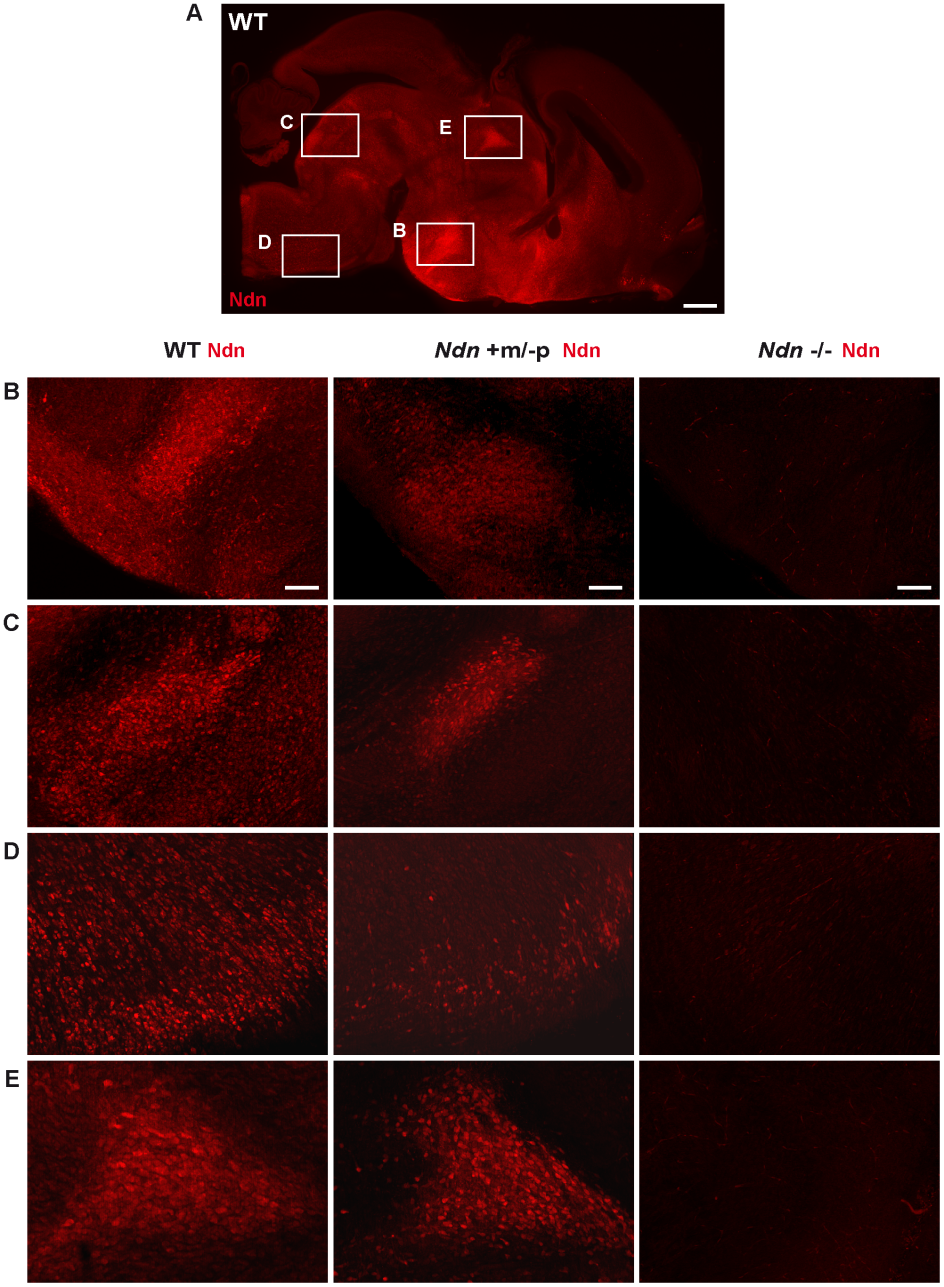
*Ndn* expression in *Ndn+m/−p* brains of neonates. Presence of Necdin in WT, *Ndn+m/−p* and *Ndn*−/− brains of neonates (P0) revealed by IHC on sagittal sections using an anti-Necdin specific antibody (red). Immunolabeling on a WT whole brain sagittal section illustrates the ventral expression of Necdin (A). Such expression is compared between the three genotypes at the dorsal medial nucleus of the hypothalamus (B), the dorsal and magnus raphe nuclei (C and D) respectively and the paraventricular thalamus nuclei (E). Scale bar: 500 µm (A) and 100 µm (B,C,D,E).

At E12.5, using immunohistochemistry (IHC) and *in situ* hybridization (ISH) on frozen serial sections, Necdin protein and transcripts were detected in the same structures of four out of nine *Ndn+m/−p* embryos (Figure 2). Importantly, no protein or transcripts were detected in *Ndn−/−* individuals (Figure S2). Expression of the maternally inherited *Ndn* allele was detected in a restricted number of cells of specific nervous structures in which the paternally inherited *Ndn* allele is normally expressed in WT animals (Figure 2 A,B,C,D,E). Interestingly, the cerebral cortex, the tongue and the myotome, which show expression of the paternal allele in WT, do not express the maternal *Ndn* allele (data not shown). In contrast to WT E10.5 embryos, no maternal *Ndn* expression was detected in *Ndn+m/−p* E10.5 embryos (n = 9) (Figure S3). At P1, a stage when expression normally peaks [24], we detected Necdin protein by IHC in the brain of *Ndn+m/−p* newborns (n = 6) (Figure 3). Necdin presented a similar expression pattern in *Ndn+m/−p* adults (Figure S4). At both developmental stages, this expression was restricted to a limited number of cells in several, but not all nuclei that express *Ndn* in WT animals, such as the hypothalamic (Figure 3B) and the raphe nuclei (Figure 3C,D).

Thus, at the anatomical level, using ISH and IHC, we conclude that the *Ndn* maternal allele is expressed in a subset of *Ndn+m/−p* individuals and, compared to WT mice, is restricted to a limited population of cells in specific nervous system structures. Noticeably, there is considerable inter-individual variability, even between littermates (Figure 1 and data not shown).

**Figure 4:**
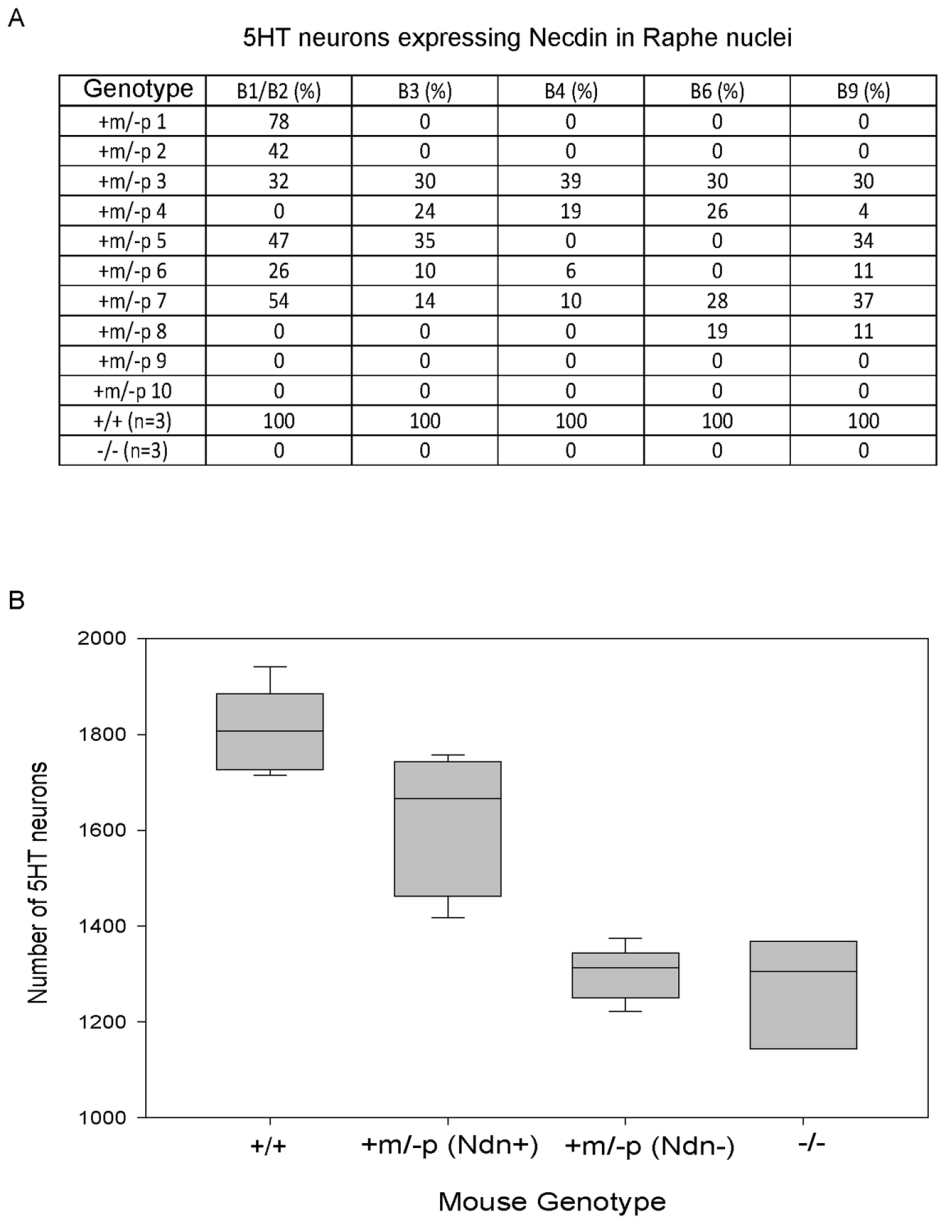
*Ndn* expression in the 5HT raphe nuclei of *Ndn+m/−p* individuals. (A) % of Necdin immunoreactive cells among 5HT positive cells located in the B1 to B9 raphe nuclei. Wild-type (n = 3), *Ndn*−/− (n = 1) and *Ndn+m/−p* (n = 10) newborn mice were analyzed. (B) Number of 5HT-expressing neurons in the B1/B2 raphe nuclei of +/+ (n = 9), *Ndn +m/−p* (n = 18) and *Ndn−/−* (n = 8) individuals. *Ndn+m/−p* (n = 18) individuals are divided in two populations: a population in which Necdin immunolabeling is detected in a mean of 46% of 5HT neurons of *Ndn+m/−* (Ndn+, n = 9) individuals and a population in which no Necdin/5HT colabelling is detected in the B1/B2 raphe nuclei of *Ndn+m/−p* (Ndn−, n = 9) individuals. Scale bar: 10 µm.

We addressed the question of intra-individual variation by studying the raphe nuclei, a structure defined by 5HT-expressing neurons, all of which express Necdin in WT mice [22]. We double immunostained P1 brains using anti-Necdin and anti-5HT antibodies and determined the number of 5HT/Necdin positive neurons in the different raphe nuclei (B1 to B9) of WT, *Ndn+m/−p* and *Ndn−/−* newborns (Figure 4A). We confirmed both inter-individual and intra-individual variation in the number of 5HT/Necdin double positive neurons in *Ndn+m/−p* raphe nuclei. For instance, in the same individual, 78% of 5HT positive neurons in B1/B2 raphe nuclei were Necdin positive although in other raphe nuclei no Necdin expression was detected (Figure 4A). We conclude that there is also intra-individual variation in the expression of maternal *Ndn* allele in the raphe nuclei.

### *Ndn* maternal expression plays a functional role at the cellular level

Previously, we observed alterations in the 5HT system [22] in *Ndn+m/−p* mice. Here, we analyzed the cellular defects in *Ndn−/−* newborn mice (n = 8) in comparison with *Ndn+m/−p* (n = 18) and WT newborns (n = 9). Using 5HT immunolabelling, we counted the number of 5HT neurons in the B1/B2 raphe nuclei (Fig. 4B). We found a significant 28% reduction (WMW test, P<0.001) in the number of 5HT-expressing neurons between *Ndn−/−* (1306 (1204,1337); n = 8) and WT newborns (1807 (1738, 1882); n = 9). Interestingly, in the B1/B2 raphe nuclei compared to WT mice, the *Ndn+m/−p* individuals that expressed Necdin, with a mean of 46% of 5HT neurons Necdin-positive (*Ndn+m/−p* (Ndn+), Figure 4B), had only a 8% reduction (WMW test, P<0.001) in the number of 5HT-expressing neurons (1666 (1489,1733); n = 9). In contrast, the *Ndn+m/−p* individuals (*Ndn+m/−p* (Ndn−), Figure 4B) that do not show Necdin expression had a significant 28% reduction (WMW test, P<0.001) in the number of 5HT-expressing neurons (1313 (1258,1335); n = 9) similar to the results observed in *Ndn−/−* P0 mice. Thus expression of the maternal *Ndn* allele in *Ndn+m/−p* individuals correlates with an increased number of 5HT-expressing neurons.

### The *Ndn* C57Bl/6J maternal allele is never expressed in wild-type mice

We next asked whether the low level of maternal *Ndn* expression was also present in WT mice. In order to discriminate between paternal and maternal allele-specific *Ndn* expression in WT mice, we identified mouse strains carrying transcribed polymorphisms in the *Ndn* gene. Three such polymorphisms (two SNPs in the 3′-untranslated region (UTR) and one 5bp indel in the 5′-UTR) were identified between *Mus musculus* (C57BL/6J) and *Mus spretus* strains.

First, to analyze the SNPs, we performed two quantifications of allele-specific expression by pyrosequencing (QUASEP) assays on RT-PCR products from F1 brains of six pups with a C57BL/6J mother and *Mus spretus* father, and did not detect expression of the maternal (C57BL/6J) *Ndn* allele in these brain samples (data not shown). To further increase the sensitivity for detection of maternal *Ndn* transcripts, we designed specific TaqMan probes distinguishing between the presence and absence of the 5 bp indel in the 5′-UTR and used RT-qPCR for allele-specific quantification. However, this assay also did not reveal any *Ndn* transcripts from the C57BL/6J maternal allele in (C57BL/6J×*Mus spretus*) F1 brains from 32 pups (Figure S5). We conclude that in this wild-type mixed genetic context, we do not detect any expression of the maternal C57Bl/6J *Ndn* allele.

### Competition between the promoters of the *Ndn* alleles

The *Ndn+m/−p* heterozygous mice described by Gerard et al [9] (named Ndn^tm2Stw^) present a more severe phenotype with in particular a higher lethality at birth compared to *Ndn^tm1.1Mus^+m/−p* mice. We failed to detect expression of the *Ndn* maternal allele in *Ndn^tm2Stw^+m/−p* embryos (n = 8) at two developmental stages (E12.5 and E14.5), using the IHC and ISH approaches (Figure S6 and data not shown). Importantly, in Ndn^tm2Stw^ mice, the *Ndn* coding sequence has been replaced by the *β-Galactosidase* sequence, and the *Ndn* promoter and regulatory sequences have been retained allowing *β.Gal* expression from the paternal allele [9]. In contrast, in *Ndn^tm1.1Mus^* mice, the promoter and the first two thirds of the *Ndn* coding sequence were replaced with a loxP site. The complete lack of *Ndn* maternal expression in the *Ndn^tm2Stw^+m/−p* mice could suggest that presence of the active paternal *Ndn* promoter suppresses expression of the *Ndn* maternal allele. We propose that the *Ndn* maternal allele is expressed only when the paternal promoter is absent or silenced, consistent with the absence of expression of the maternally inherited *Ndn* allele in WT mice (Figure S10A).

**Figure 5:**
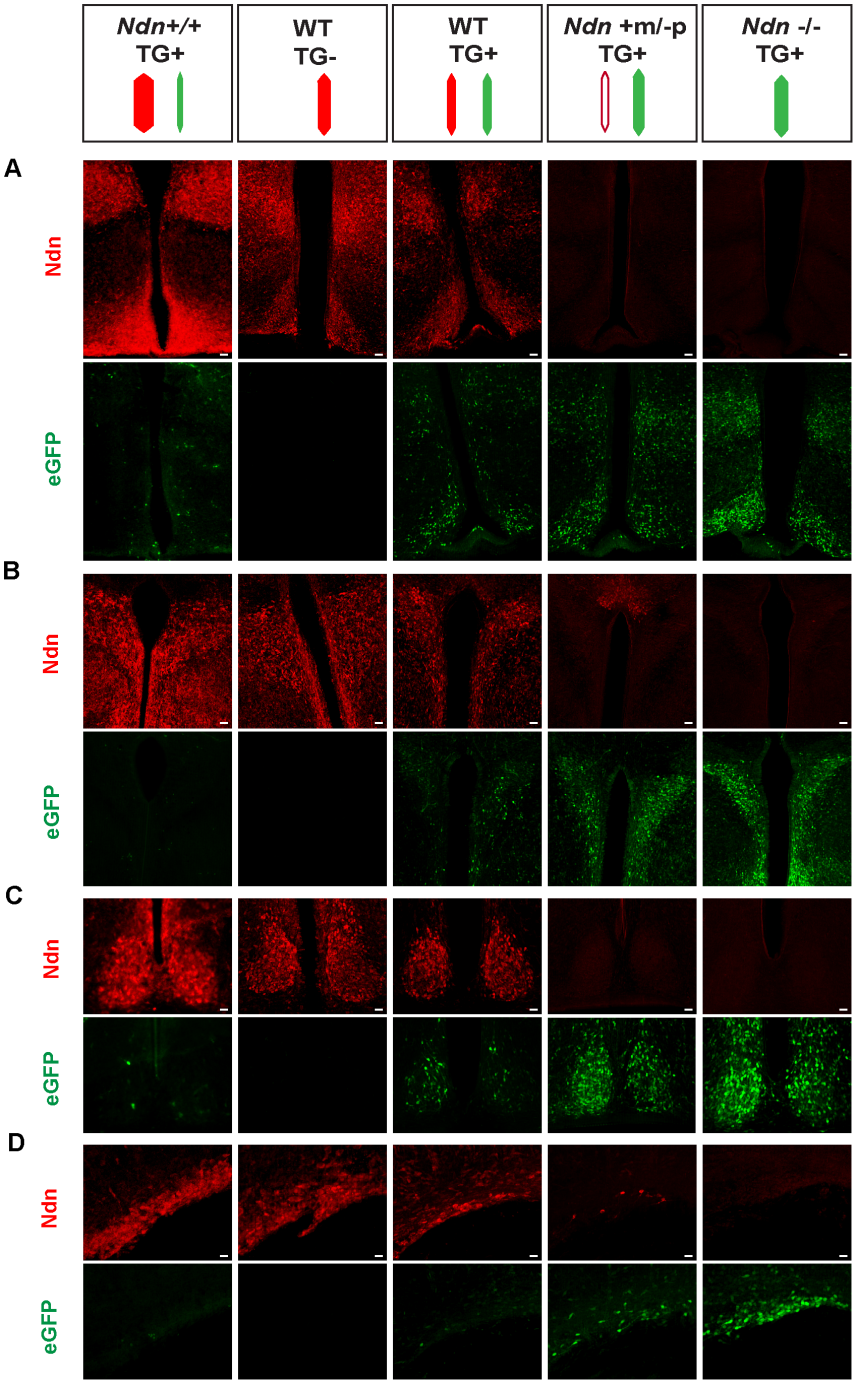
Necdin expression and eGFP expression in different mouse genetic backgrounds with or without the BAC *Ndn*-eGFP transgene (TG+ or TG−). WT(TG−), WT(TG+), Ndn++(TG+), *Ndn+m/−p*(TG+) and *Ndn−/−*(TG+) mice are analyzed by IHC to detect Necdin or eGFP expression. The coloured dashes indicate: *Ndn* maternal allele (white), *Ndn* paternal allele (red) and BAC TG (green); the width of the dashes indicates the level of expression of Ndn or eGFP. Immunostaining for Necdin (red) and eGFP fluorescence (green) was performed on coronal cryosections of brains at P0. The expression was studied in several hypothalamic nuclei: dorsomedial hypothalamus and arcuate (A), paraventricular (B), suprachiasmatic (C) and supraoptic (D). Interestingly, in a *Ndn+m/−p* (TG+) hypothalamus with a faint expression of the maternal allele, we also detect fewer eGFP-positive cells than in a *Ndn−/−* (TG+) hypothalamus. Scale bar: 20 µm.

To further explore this question, we created a transgenic mouse line (TG45 named TG) containing a modified Bacterial Artificial Chromosome (BAC) in which the *Ndn* coding sequence was replaced by the *eGFP* sequence under the control of the *Ndn* promotert; this BAC transgene is present in one or two copies and its expression is not regulated by imprinting mechanism (Figure S7 and data not shown). In these mice, the expression of eGFP was restricted to the brain regions in which *Ndn* is normally expressed (Figure 5 and data not shown). We then studied this eGFP expression in newborn WT, *Ndn+m/−p*, *Ndn−/−* hypothalamus (using the *Ndn^tm1.1Mus^* strain) and in the hypothalamus from a mouse line (Ndn*++*) in which *Ndn* is over-expressed (Figure 5 and Figure S10B). We performed colabelling and observed an inverse correlation between the intensity of Necdin immunolabeling and eGFP fluorescence on coronal brain sections (Figure 5). In the absence of *Ndn* expression (*Ndn*−/−, TG+) the number of eGFP-positive cells was the highest compared to eGFP-positive cells when *Ndn* is over-expressed (*Ndn++*, TG+) (Figure 5). This result was quantified by immunoblotting (Figure S8A). Finally, in (WT, TG+) mice, we independently quantified for each cell the eGFP and Necdin signals in two hypothalamic nuclei (Figure S8 B,C). For both structures, we observed two distinct populations of cells and conclude that approximately half of the cells expressed Necdin while the other half expressed eGFP; only very few cells co-expressed eGFP and Necdin (Figure S8D). Thus, at the cellular level, the eGFP expression level is inversely correlated with Necdin expression level.

Our results are consistent with a model whereby two *Ndn* alleles in the same cell, both of which include at least the *Ndn* promoter and regulatory sequences and neither of which is silenced by imprinting, triggers allelic exclusion at the transcriptional level favouring the expression of one allele only per cell (Figure S10).

### Analysis of the methylation profile at the *Ndn* DMR

Variation in DNA methylation at the DMRs of imprinted genes has been reported in different tissues, importantly in brain, and might be a source of gene expression and phenotypic variations [25]. We therefore studied DNA methylation in a secondary DMR (42 CpGs), previously shown to be correlated with imprinted regulation of *Ndn* expression [7], [26] (Figure S9). We found no major changes in methylation on the *Ndn* maternal allele in *Ndn+m/−p* brains. However, methylation of this DMR occurs after the blastula stage and our failure to detect modifications to methylation in this DMR could be because only a few neurons express the maternal *Ndn* allele, and this escapes our global brain analysis.

### The maternally inherited allele of *NDN* is expressed in hypothalamus of PWS patients

**Figure 6:**
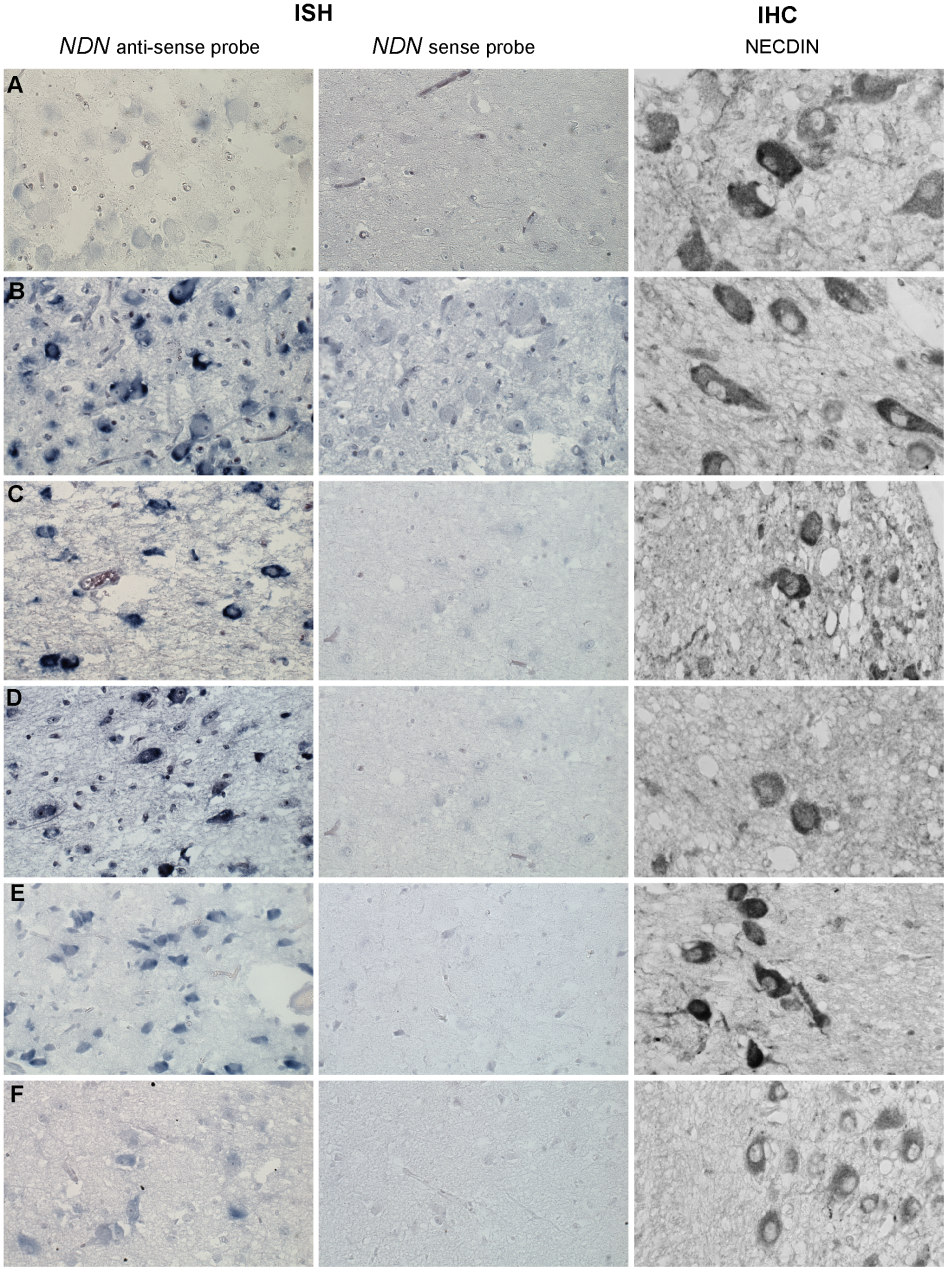
*NDN* expression in PWS patients. Detection of *NDN* transcripts revealed by ISH, and using a *NDN* anti-sense probe, on PVN brain sections from control individuals (A,C,E) and PWS patients (B,D,F). A *NDN*-sense probe was used as a negative control. IHC on SON brain sections, using a NECDIN specific antibody, was performed on the same control and PWS patients. The expression was studied in the 94-118 adult control male (A) and the 95104 adult PWS patient with a maternal uniparental disomy (B), in the 88-017 adult control male (C) and the 00-028 PWS adult patient with a deletion (D), in the 97-153 control infant (E) and the 99-079 PWS infant with a deletion (F). Scale bar: 20 µm.

We assessed whether the expression of the maternal *Ndn* allele observed in heterozygous *Ndn+m/−p* mice also occurs in PWS patients. Using a specific human *NDN* RNA probe and an anti-Necdin antibody, we performed an ISH and IHC on hypothalamic sections obtained from brains from two adult PWS patients (one with a deletion and one with a maternal disomy) and one PWS infant (9 months old with a deletion); age and sex matched control individuals were included as positive controls (Table S1). In all patients, we found *NDN* transcripts and protein in the paraventricular and supra optic nuclei (Figure 6). We confirmed *NDN* mRNA expression in five more adult PWS patients (25–64 years of age) and one PWS child (6 months old). Expression of *NDN* also occurred in the cortex of PWS patients (data not shown). The results contradict the widely accepted assumption that in PWS patients the maternal allele is totally silenced in the brain. These findings are in full agreement with the results obtained in our heterozygous *Ndn+m/−p* mice.

## Discussion

In this article we report a stochastic expression of the maternally inherited allele of the *Ndn* gene in mice where the paternal gene has been inactivated. We showed an extremely low and very variable number of transcripts but nevertheless confirmed that these transcripts are translated into Necdin protein. Furthermore a comparison between *Ndn−/−* and *Ndn+m/−p* pups showed that the lethality, due to respiratory distress, is decreased two-fold in *Ndn+m/−p* compared to *Ndn−/−*. In agreement with this decreased lethality, surviving *Ndn+m/−p* adult mice present two-fold fewer apneas and more 5HT-expressing neurons, in a manner that is positively correlated with maternal *Ndn* expression. This confirms the functional importance of the extremely weak expression of the *Ndn* maternal allele. Finally *NDN* transcripts and protein were also detected in brain tissue from human PWS patients.

Furthermore, our results strongly suggest that expression of the maternal allele of *Ndn* only occurs in the absence of expression of the paternal *Ndn* allele. In addition, they are consistent with a model where, without an imprinting regulation, competition between two *Ndn* promoters results in a monoallelic expression. In this model, imprinting mechanisms create an allelic exclusion that dictates that the maternal allele is inactivated.

### Loss of imprinting in mouse models for PWS and in PWS patients

Prior to this study, it was widely accepted that only the paternal alleles of PWS candidate genes are expressed, the maternal alleles being totally silenced. However, in brains of mice, with a deletion of the imprinting center, an incomplete silencing of paternally inherited PWS genes as well as a low level of expression of maternal alleles of PWS genes, was reported but not investigated [27]. LOI was also observed in lymphoblasts of two PWS patients with a deletion and two atypical PWS patients with a maternal disomy [28], [29], but these studies were not extended to include expression profiles in the brain. LOI has been described in other contexts, particularly in some cancers [30]. Our study addresses for the first time the robustness of silencing of the maternal alleles of PWS candidate genes in brain. Our results show that in both mice and humans, in the absence of the paternally inherited *Ndn* gene, the maternal *Ndn* allele is expressed in the brain at very low level but sufficiently to allow Necdin protein production. A similar mechanism might be hypothesized for any of the PWS genes in PWS patients. For example, the imprinted *Magel2/MAGEL2* PWS gene, showed a similar loss of imprinting in *Magel2+m/−p* heterozygous mice and PWS human brains (F.M. and D.S. unpublished data). Significantly, the high variability of expression of maternal alleles of these genes might explain the large degree of heterogeneity in the severity of PWS symptoms.

### The relevance of *Ndn* maternal allele expression

The two-fold reduction of post-natal mortality in *Ndn+m/−p* mice compared to *Ndn−/−* mice, suggests that even the low level of maternal Necdin protein is sufficient to rescue 50% of the mice, in comparison with the *Ndn−/−* mice. Nevertheless 50% of *Ndn−/−* individuals survive suggesting that another compensatory system is activated when the level of *Ndn* expression is null or very low in *Ndn+m/−p* mice.

A surprising degree of inter-individual variability was observed in the number of *Ndn* transcripts amongst *Ndn+m/−p* mice. The degree of maternal *Ndn* expression is correlated with the severity of the phenotype, in that the number of apneas is significantly increased in *Ndn−/−* mice compared *to Ndn+m/−p* mice. Previously, we published that those apneas might be correlated with an alteration of the 5HT system [22]. Here we showed that the number of 5HT-expressing neurons is reduced by 28% in *Ndn−/−* compared to WT mice while *Ndn+m/−p* mice are divided in two distinct populations with 30% and 10% fewer 5HT-neurons respectively. The lowest reduction (10%) of 5HT-expressing neurons is observed in those *Ndn+m/−p* individuals co-expressing Necdin in 5HT neurons. These data support a link between the expression of the *Ndn* maternal allele and the degree of survival, the severity of apneas and the number of 5HT neurons in the B1/B2 raphe nuclei.

### Variability of *Ndn* maternal expression

*Ndn* maternal expression presents a high inter-individual variability (1 to 3 orders of magnitude), even among *Ndn+m/−p* individuals from the same litter, irrespective of the age and brain structure analyzed. Intra-individual variability of *Ndn* expression was also detected in the brain structures. This expression is limited to some, but not all, of the brain regions that normally express *Ndn* with no evidence of ectopic expression. In those brain regions the number of neurons expressing *Ndn* is clearly less than in wild-type animals and variability of *Ndn* expression amongst the *Ndn+m/−p* offspring was linked to both maternal genotype and gender, being additive factors. A *Ndn+m/−p* mouse with a +/+ or *Ndn+m/−p* mother (a mouse who has inherited a wild-type *Ndn* allele from her grandmother) is predisposed to the highest level of expression and to a greater inter-individual variability, a phenomenon referred to as transgenerational epigenetic inheritance [31]. In contrast, paternal genotype has no impact. Furthermore maternal *Ndn* allele expression was two-fold higher and more variable in female mice compared to male mice. This may reflect the increased genetic variability in females: some genes escaping X inactivation, such as *Jarid1c*, which codes for a histone demethylase [32], showing higher expression in females. This could explain our observations concerning maternal allele *Ndn* expression. Alternatively or additionally, female-specific hormones could be involved.

An interesting observation resulting from transcriptome profiling is the very high variability between individuals in steady state levels of a range of mRNAs, often reaching an order of magnitude [33]. This might explain why the penetrance of a given genotype is often incomplete [23], [31], [33]. This type of epigenetic phenomenon might also be involved in the variable expression of the maternal *Ndn* gene and consequently might lead to survival of some *Ndn+m/−p* mice. Nevertheless, even in *Ndn*−/− mice the penetrance of the phenotype (postnatal lethality and apneas) is not complete, suggesting that another mechanism involving a “compensatory pathway” takes place. This compensatory pathway might result from an increase of a gene expression linked to the lack of Ndn expression or might also result from the stochastic variability in gene expression described above [33] that occurs independent of the state of Ndn expression.

### Mechanism underlying the maternal expression

The lack of detection of expression of the C57Bl/6J maternal allele in WT mice (with a paternal M. Spretus allele) suggests that expression of the *Ndn* maternal allele is associated with the absence of an active paternal *Ndn* promoter, as confirmed by our study of the *Ndn+m/−p* Ndn^tm2Stw^ embryos that did not express the maternal allele of *Ndn*. Similarly, in a third *Ndn*-KO mouse model, in which the *Ndn*-promoter also drives β-gal expression [18], no maternal *Ndn* transcripts were detected by RT-qPCR [34] in *Ndn+m/−p* mice. Previously, Chamberlain et al. observed a low level of *Ndn* maternal expression only in the absence of an active paternal PWS-imprinting center, and suggested a *trans* effect where the paternal PWS-IC acts on the maternal allele [27]. Furthermore, our data suggest that, even in the absence of imprinted regulation of *Ndn*, as is the case for the *Ndn-eGFP* BAC transgene, it appears that there is a transcriptional regulation predisposing to a monoallelic expression of *Ndn*. This result might be explained by promoter competition for transcriptional activators, or a mechanism involving physical contact in *trans* between promoters [27], [35]–[37].

### Expression levels of *Ndn* mRNA and protein

Given the extremely low level of *Ndn* transcripts in +m/−p mice as estimated by RT-qPCR it is surprising that the protein was detectable by immunohistochemistry and by Western blot. Importantly, the absence of antibody staining on samples from −/− mice ruled out the possibility of cross-reactivity with proteins sharing epitopes with Necdin. Until relatively recently, it has been assumed that transcript abundance is the main, although not the only, determinant of protein abundance. Experiments aimed at addressing this question have lead to an emerging body of evidence changing this view and, in every organism that has been examined to date at a global level, steady-state transcript abundance only partially predicted protein abundance [38]. This lack of correlation suggests a strong regulatory role for all processes downstream of transcription. Furthermore, it has been shown that in many situations, transcription, translation and degradation are often extensively coupled and regulate each other through feedback loops. This coupling might enhance responsiveness to the environment and might help reduce inter-cellular variability in gene expression, which is by nature a stochastic event [39].

Collectively, these results suggest that very low level expression of PWS maternally silenced genes might be sufficient to alleviate specific PWS symptoms [23]. Importantly, we show that the quantity of *Ndn* transcripts is not, at least in neurons, a good indicator of its protein level and hence its functional importance [38].

An understanding of the context in which the *Ndn* maternal allele might be transcribed is an important step towards the development of a pharmacological therapy to trigger and/or increase the expression of this maternal allele in PWS patients. Furthermore, our results provide a further indication of the high non-genetic heterogeneity between genetically identical individuals that might, in this case, underlie LOI and contribute to variability in the phenotype [40].

## Materials and Methods

### Breeding of mice

Mice were handled and cared in accordance with the *Guide for the Care and Use of Laboratory Animals* (N.R.C., 1996) and the European Communities Council Directive of September 22th 2010 (2010/63/EU, 74). Experimental protocols were approved by the institutional Ethical Committee guidelines for animal research with the accreditation no. B13-055-19 from the French Ministry of Agriculture.

*Ndn* deficient mice were maintained on the C57BL/6J background and the paternal mutation was transmitted by crossing *Ndn*−m/+p males with C57BL/6J WT females (from Janvier Company). In parallel, since the *Ndn*-KO allele was created using a 129/SvPas ES cell line, we maintained the mutation via a maternal transmission on the 129/SvPas genetic background using Charles River male mice. All *Ndn* mice were genotyped by PCR as previously described [21]. Genotype of *Ndn−/−* mice was confirmed by a secondary intra-deletional PCR whose primers were: 5′-GATCCGAAGGCGCAGACATG-3′ and 5′-CTGCCCATGACCTCTTTCAC-3′ generating a 420 bp fragment indicating the presence of *Ndn* WT allele.

The *Ndn ++* over-expressing mouse line is the *Magel2* KO (+m/−p and −/−) mouse line created previously in our team. Consequently, it is an over-expression of the endogenous *Ndn* gene rather than being a transgenic mouse. *Magel2* being imprinted, closed to *Ndn* and belonging to the MAGE family gene, as Ndn. We observed this overexpression at the transcript and protein level and we estimated, by western blot quantification, the level of overexpression (a factor of 1.7 fold). They were also maintained onto C57BL/6J background.

Importantly, all the mouse lines used in this study, excepted the *Ndntm2Stw*+m/−p mouse line, have been created in our laboratory and maintained on pure genetic background. The *Ndntm2Stw*+m/−p mouse have also been bred onto C57Bl/6 for over 30 generations in Wevrick's laboratory.”

### Western blotting

P1 brain and E12 whole embryos were rapidly dissected and crushed in lysis buffer as previously [41]. For each sample, proteins (30 mg) were separated on a 12% SDS-PAGE and transferred onto nitrocellulose membranes (Protran Whatman, Dutscher). Membranes were incubated overnight with a rabbit polyclonal antibody against Necdin (Upstate; 1∶1000) or with a rabbit polyclonal antibody against GFP (Sigma, G1544) and subsequently with an anti-rabbit horseradish peroxydase antibody (GE Healthcare, Buckinghamshire,UK; 1∶3000). In both experiments, membranes were reprobed using a mouse anti-α-Tubulin antibody (Sigma, T6074). For Necdin immunolabeling was visualized by enhanced chemiluminescence. For GFP immunolabeling was visualized by Gbox (Syngen). Quantification was performed using ImageJ.

### Plethysmography

We performed plethysmography in weight-matched littermate mice that were 6 weeks old, unrestrained and unanesthetized. Spontaneous breathing activities were recorded in normoxic conditions using whole-body plethysmograph (EMKA Technologies, Paris, France). After a 30 min period of stabilization in the apparatus, respiratory parameters were calculated breath-by-breath during a 30 min period of measurement. The mean of each parameter was automatically calculated from this 30 min period of measurement using EMKA technologies Datanalyst software. Apneas have been defined here as an absence of a respiratory signal during at least three respiratory cycles in resting conditions.

### Reverse transcription and real-time quantitative PCR

Classical RT-PCR was performed as previously described [17].

For RT-qPCR, mice were sacrified at E12.5, P1 or as adults. Whole embryos, whole P1 brains, P1 pons and P1 or adult hypothalamus tissues were rapidly collected and frozen in liquid nitrogen prior to RNA isolation using standard conditions. Subsequently, total RNA samples were incubated with DNase (TURBO DNA-free; Ambion). Messenger RNAs from 1 µg of total RNAs were reverse-transcribed in a total volume of 20 µL using the M-MLV, reverse transcriptase RNAse H minus, point mutant (Promega) and oligod(T)_15_ in the presence of a synthetic external, heterologous and noncompetitive poly(A) Standard RNA (SmRNA) used to calibrate the reverse transcription [42] (patent WO2004.092414). At the end of the RT, total volume was brought up to 100 µL and real-time PCR was performed using the Rotorgene System (Qiagen) to determine the number of SmRNA and *Ndn* cDNA molecules in 5 µL of the RT product. The specific forward and reverse primers were designed using “Universal Probe Library” software (Roche Diagnostics) in the region deleted in the *Ndn* KO-allele. The sequences of the primer pair used were: Necdin-Forward 5′-AACAACCGTATGCCCATGA-3′, Necdin-Reverse 5′-CTTCACATAGATGAGGCTCAGGAT-3′ (60 bp). The primer sequences and the quantification conditions of calibrator cDNAs (Standard cDNAs) are protected by the patent WO2004.092414. To discriminate specific from nonspecific cDNA products, a melting curve was obtained at the end of each run, by a slow temperature elevation up to 98°C (0.1°C.s-1). Before RT, absence of traces of genomic DNA in the purified total RNA samples was ruled out by real-time PCR of the non-deleted *Ndn* sequence. Quantification cycles were converted into the number of cDNA copies using the quantification curve specific for each primer pair that had been previously established from serial dilutions of purified PCR products. The equation of the calibration curve for NDN cDNA was performed in four replicates for each dilution ranging from 10 to 1×10^9^ copies : C_t_ = −3.3417 Log [cDNA]_i_+39.049, r^2^ = 0.9988. No amplification was obtained in *Ndn*−/− individuals only. In the other mice, the lowest and highest copy numbers quantified were 92 and 4,638,062, respectively. For each sample, the number of *Ndn* cDNA copies was normalized according to relative efficiency of RT determined by the standard cDNA quantification. Finally, gene expression was expressed as the cDNA copy number quantified in 5 µL aliquot of RT product.

### Immunohistochemistry

Specificity of Necdin protein detection was controlled on tissues from *Ndn−/−* animals. Fixed brains was dissected, cryopreserved and sectioned (14 µm) using a cryostat (Leica CM3050S). Embryos and post-natal mice (P1) were sacrificed and treated as previously [21]. Antibodies used were: rabbit polyclonal anti-Necdin (07-565; Millipore, Bedford, MA, USA; 1∶500), mouse monoclonal anti-GFP (Interchim, NB600-597; 1∶500), goat polyclonal anti-5HT (Immunostar, 20079; 1∶300).

Sections were washed twice in PBS and incubated with Hoechst (33258, Sigma; 1∶2000) and corresponding fluorochrome-conjugated secondary antibodies, goat anti-rabbit Alexa Fluor 488 or Alexa Fluor 555 (Molecular Probes, Invitrogen; 1/500), goat anti-mouse Alexa Fluor 488 (Molecular Probes, Invitrogen; 1/500), donkey anti-goat Cy3 (Chemicon, AP180C; 1/1000) diluted in the blocking buffer without BSA. Sections were examined on a Zeiss Axioplan 2 microscope with an Apotome module. Quantification of labeled cells was performed using ImageJ.

For quantification of immunofluorescence, images were acquired using a confocal microscope (SP5-X, Leica), z stacks of 70 µm were performed for each image, and analyzed using ImageJ.

### *In Situ* hybridization

All *Ndn in situ* hybridization experiments for the study of *Ndn* gene expression were performed on serial slices of those used for immunohistochemistry and performed as previously [43]. Specificity of *Ndn* mRNA detection was controlled on tissues from *Ndn−/−* animals and with the sense control riboprobes. A peroxidase-conjugated anti-digoxigenin-POD (1∶1250) antibody (Roche) was used to detect the *Ndn* hybridized riboprobe, visualized using a tyramide signal amplification (TSA-plus Biotin Kit, Perkin Elmer).

### Quantification of allele-specific expression

We could identify two transcribed Single Nucleotide Polymorphisms (tSNPs) in the 3′-UTR of *Ndn* to discriminate between the C57BL/6J and *Mus spretus* alleles.

To determine allele-specific transcription levels, we performed QUASEP and RT-qPCR with allele-specific TaqMan probes on cDNA of C57BL/6J×*Mus spretus* F1 brains from P10–P14 pups. All RNA samples were treated with DNaseI (Agilent) to minimize any risk of contamination with genomic DNA. Subsequently, 2 µg of high-quality total RNA were reverse transcribed into cDNA (SuperScript III First Strand Synthesis System, Invitrogen) and oligo(dT)-priming according to manufacturer's instructions.

The QUASEP assays were designed using the PyroMark Assay Design Software 2.0 (Qiagen). PCR was performed with the FastStart High Fidelity PCR System (Roche) according to manufacturer's recommendations using the cDNA of C57BL/6J×*Mus spretus* F1 brains from 6 P10–P14 mice. Pyrosequencing was done on a PSQ 96MA Pyrosequencing System (Qiagen) with a sequencing primer (Table S2) and PyroGold SQA reagents (Qiagen). Data were analyzed with the PSQ 96MA 2.1.1 software (Qiagen) as previously described [44].

For allele-specific RT-qPCR, we used the 5 bp indel in the 5′-UTR of *Ndn* to design TaqMan probes specific for C57BL/6J and *Mus spretus*, respectively [45], [46]. Quantitative PCR was performed on an ABI 7500 Fast Real time PCR System using the cDNA of C57BL/6J×*Mus spretus* F1 brains from 32 P10–P14 mice. Briefly, the 20 µl reaction contained 10 µl TaqMan Fast Universal PCR Master Mix (2×), 3.6 µl 5 µM combined forward (C57BL/6J: 5′-CTTCCTCTGCTGGTCTCCAC-3′, Mus spretus: 5′-CTTCCTCTGCTGGTCTCCAC-3′) and reverse (C57BL/6J: 5′-GGGTCGCTCAGGTCCTTACT-3′, Mus spretus: 5′- GGGTCGCTCAGGTCCTTACT-3′) primers (0.9 µM); 2 µl of each 2 µM TaqMan probe (C57BL/6J: FAM-CTCCAAGCCGCATCGGTCCTGCTC-BHQ1, Mus spretus: ATTO550-CTCCAAGCCGCATCGCATCGGTCC-BHQ2; 0.2 µM) and 2.4 µl cDNA. The qPCR thermal profile consisted of 95°C for 10 min, followed by 48 cycles of 95°C for 30 s and 60°C for 30 s. Real-time PCR data were analyzed with ABI SDS 2.0.6 software.

### Methylation study

Unfertilized oocytes and blastocysts were collected from C57BL/6 superovulated females and directly embedded in agarose beads for bisulphite treatment as previously described [47]. Sperm was recovered from the epididymis. Adult brain and kidney were dissected from interspecific M. spretus X M. musculus F1 mice or from *Ndn+m/−p* mice and DNA extracted according to standard techniques.

Bisulphite treatment of HindIII-digested adult brain, kidney and sperm genomic DNAs was carried out as described [48]. Oocyte and blastocyst DNAs were treated as described [47]. A semi-nested PCR was used to amplify regions A (CpG sites 1 to 20) and B (CpG sites 21 to 42) from bisulphite treated DNA samples. Primers used to amplify region A were: 5′-TGTGTTATATAGGAGATTAGG-3′ (outside forward; first and second rounds), 5′-AAACTACCATAAAACCTT-3′ (outside reverse) and 5′-CTATCCTACATCTCACAA-3′ (inside reverse). Primers used to amplify region B were: 5′-ATTGTGAGATGTAGGATAG-3′ (outside forward; first and second rounds), 5′-CCATAACCTCTTTCACCATA-3′ (outside reverse), and 5′-AAACTACCATAAAACCTTC-3′ (inside reverse). PCRs were performed in 50 µl reactions containing 1.25% DMSO, 25 pmole of each primer, 0.2 mM dNTPs, 2.5 mM MgCl2, 1× PCR Buffer and 2.5 U Q-BioTaq DNA Polymerase (Quantum Appligene, Germany). Second round PCRs were performed using 1 µl of the purified primary PCR products. PCR cycles were 5 min at 94°C followed by 10 cycles of 30 s at 94°C, 30 s at 58°C, 25 s at 72°C, and by 25 cycles of 30 s at 94°C, 30 s at 58°C, 25 s plus 5 s at each cycle at 72°C, and 7 min at 72°C. Purified PCRs products were cloned into the pGEM-T Easy TA Vector (Promega) and sequenced using standard methods. For oocytes and blastocysts, PCRs were performed on samples prepared from at least three different batches of oocytes and blastocysts. Identical clones derived from oocytes and blastocysts secondary PCRs were considered as derived from one single allele and represented only once.

### Generation of transgenic mice with *Ndn*-*eGFP* modified BAC

The BAC*603M20* (Research Genetics; referred as BAC109 [43]) contains a 104 kb *NotI* insert including the *Ndn* gene. BAC109 was modified by homologous recombination in E.coli as described [49] in order to replace the *Ndn* open reading frame (ORF) by the *eGFP* ORF.

Cesium chloride gradient purified BAC DNA was microinjected in the pronucleus of C57BL/6× CBA mouse zygotes. Founders containing the BAC transgene were identified by amplifying an eGFP fragment by PCR. Transgenic founders were maintained on C57BL6 genetic background. Transgene copy numbers were determined by Southern blot of *BglII* digested genomic DNA hybridized with PCR probes.

### Human material and studies

Hypothalamic material from 3 PWS patients and from controls, matched for age, sex, postmortem delay and fixation time were obtained through The Netherlands Brain Bank (NBB, Director Dr. I. Huitinga). Clinicopathological details are given in Supplementary Table S1.

Sections throughout the hypothalamus were collected at 1200 µm intervals and mounted and pretreated as previously [50], with 2 µg/ml of proteinase K.

For the detection of *NDN* mRNA we hybridized the sections with a 2000 ng/ml DIG-labeled RNA probe, complementary to bp1258–1578 of the human *NDN* mRNA (NM_002487.2). Hybridization and stringency washes were performed as previously described [51] at 60°C. Anti-DIG-Alkaline phosphatase-fab fragments (Roche), diluted 1∶3000 in buffer 1 (100 mM Tris, 150 mM NaCl pH 7.5) were used to detect DIG labeled RNA hybrids [50]. Specificity of the hybridization signal was verified by comparison with sections processed with sense probe under identical conditions.

For IHC, 6 µm sections of hypothalamus tissue, containing SON, PVN, and INF were collected, mounted and microwaved in Citrate Buffer as previously [50]. Necdin was detected with rabbit IgG, anti-Necdin (07-565; Millipore, Bedford, MA, USA) diluted 1∶500 in Supermix (SUMI: 0.25% gelatin (Merck) (w/v), 0.5% Triton X-100 in TBS, pH 7.6) for 1 hour at RT, followed by an overnight incubation at 4°C. Detection of Necdin immunoreactivity was performed according to the ABC method described before [52]. Antibody specificity was confirmed by the absence of ICC staining in the human hypothalamus after omission of the first antibody from the staining protocol.

### Statistical analyses

Nonparametric statistical tools (Sigmastat software) or exact statistical tools (StatXact software) were used depending on the size of the sample (n). All tests are two-tailed tests. In the results, values are indicated as following: Mean±SD or (Q2 (Q1, Q3), n, P value) where Q2 is the median, Q1 is the first quartile and Q3 is the third quartile. Mann Whitney t-test or Wilcoxon-Mann-Whitney test (WMW in the text) are used. The level of significance was set at a P-value less than 0.05.

## Supporting Information

Figure S1 *Ndn* expression analyzed by RT-PCR and Western blot. (A and B) Western-blot analysis on homogenates from P1 whole-brain (A) or from E12.5 embryos (B) using an anti-Necdin antibody and an anti-Tubulin antibody as positive control. Necdin-specific immunoreactivity (at 37 Kd) was visible in all WT and in four *Ndn+m/−p* mice, while four *Ndn+m/−p* and *Ndn−/−* mutants showed no Necdin expression (A,B). (C) RT-PCR analysis to detect a 562 bp fragment of *Ndn* transcripts in whole brain of WT, *Ndn+m/−p* and *Ndn−/−* neonates. Note the complete absence of *Ndn* expression from the maternal allele in the neonatal brain using this approach. A positive control PCR was performed using *Hprt* primers in order to amplify a 429 bp fragment of *Hprt* transcripts (C, *Hprt*).
(TIF)

Figure S2 *Ndn* expression in *Ndn*−/− E12.5 embryos. Expression of Ndn in the nervous system of WT and *Ndn*−/− embryos at E12.5 revealed by IHC or ISH on frozen sections using an anti-Necdin antibody (red) or *Ndn* RNA probe (green). Tissue sections are visualized using a Hoechst labeling (blue). Although expression is detected in WT embryos at the protein and transcript levels in the preoptic area (A), supraoptic area (B), thalamus (C), pons (D) and in the dorsal root ganglia (E), no transcripts or protein are detected in *Ndn* −/− embryos. Scale bar: 50 µm.
(TIF)

Figure S3 *Ndn* expression in *Ndn+m/−p* E10.5 embryos. Expression of Necdin in the nervous system of WT and *Ndn+m/−p Ndn* embryos at E10.5 revealed by IHC or ISH on frozen sections using an anti-Necdin antibody (red) or a *Ndn* RNA probe (green). Tissue sections are visualized using a Hoechst labeling (blue). Expression is detected in WT embryos at the protein and transcript levels in the preoptic area (A), supraoptic area (B), thalamus (C), pons (D) and in the dorsal root ganglia (E). No expression is found in *Ndn+m/−p* embryos (n = 9). Scale bar: 50 µm.
(TIF)

Figure S4 Necdin expression in *Ndn+m/−p* adult brains. Expression of Necdin in WT, *Ndn+m/−p* and *Ndn−/−* adult brains revealed by IHC using an anti-Necdin antibody (in red) on coronal sections at the lateral septum level (A), nucleus of thalamus (B), arcuate nucleus of hypothalamus (C), paraventricular hypothalamic nucleus (D) and suprachiasmatic nucleus (D). Scale bar: 500 µm (A), 250 µm (B), 200 µm (C and D).
(TIF)

Figure S5 RT-qPCR shows an absence of *Ndn* maternal allele expression in wild-type mice. TaqMan probe-based RT-qPCR analysis for allele-specific quantification of the 5 bp indel polymorphism on brain cDNA samples of *Mus musculus* (C57Bl/6J) (A), *Mus spretus* (B) and two F1 hybrid offspring from a female *Mus musculus X male Mus spretus* cross (C and D). The blue curve and the horizontal blue line represent transcripts from the *Mus musculus* allele and the corresponding threshold C_t_ value, the red curve and the horizontal red line transcripts from the *Mus spretus* allele and the corresponding threshold C_t_ value. The results obtained for the brain cDNA samples of the other 30 F1 hybrid mice were identical to those shown in C and D.
(TIF)

Figure S6 Expression of *Ndn* in the nervous system of wild-type and *Ndn+m/−p* Ndn^tm2Stw^ embryos at E12.5. We performed IHC or *ISH* on frozen sections using an anti-Necdin antibody (in red) or *Ndn* RNA probe (in green). Tissue sections are visualized using a Hoechst labeling (blue). An expression is detected at the protein and transcript levels in WT embryos only, in the septal area (A), preoptic area (B), thalamus (C), pons (D) and in the dorsal root ganglia (E). Note that there is no expression in *Ndn+m/−p Ndn^tm2Stw^* embryos. Scale bar: 50 µm.
(TIF)

Figure S7 BAC *Ndn*-eGFP transgene construction and analyses. Structure and copy number of the *603M20-deltaNdn-eGFP* transgene in line TG45 (referred in the text to TG) and TG57 (not studied here but shown for comparison to TG45). (A) The *Ndn* genomic region and (B) *603M20-deltaNdn-eGFP* transgene are represented on the upper diagram. (C) Genomic DNA was isolated from WT or transgenic mice (lines 45 and 57), digested by *Bgl*II, separated by gel electrophoresis, blotted and hybridized to the probe 1. In line 45, the 4.4 kb transgenic and the 4.9 kb endogenous fragments detected after hybridization were of same intensity, indicating that the transgene was present in 1 or 2 copies although several copies are detected in line 57. Only the TG45 mouse line expresses the eGFP transgene and was used in our experiments.
(TIF)

Figure S8 Quantitative analyses of eGFP expression and Necdin expression in the hypothalamus of WT TG+ mice. (A) Western blot analysis to quantify the eGFP expression relative to α-Tubulin in WT, *Ndn+m/−p* and, *Ndn−/−* hypothalamus of TG+ mice. The ratio of eGFP/α Tubulin (R) was calculated for each *Ndn+m/−p* individual and a mean (n = 3) is given for the WT and *Ndn*−/− genotypes. R is an indicator of the hypothalamic quantity of eGFP per genotype. (B,C) Immunofluorescence (green) of the Ndn-eGFP transgene and immunolabeling of the Necdin-positive cells (red) in the PVN (b) or the NSC (C). (D,E) Graph of the quantification of green and red fluorescence performed for each individual cell from the PVN (D) and NSC (E) brain structures.
(TIF)

Figure S9 Methylation profile at the *Ndn* DMR. (A) Localization of CpG dinucleotides in relation to the *Ndn* transcriptional start site, translational start site and promoter is represented. The transcriptional start site is between CpG sites 10 and 11, and the translational start site (ATG) between CpG sites 16 and 17. (B) and (C) CpG dinucleotides are very sparsely methylated or completely unmethylated in regions A and B of the paternal alleles in brain (B) and kidney (C). In regions A and B, the maternal alleles display a much higher level of CpG methylation, the average percentage of methylated CpG being 44% in region B in brain (D) and kidney (E) of WT mice as in brain of *Ndn+m/−p* mice (F). It should be noted that no particular bias of amplification in either region A or B of paternal versus maternal alleles was observed and that the maternal methylation profiles described were identical in interspecific *M. spretus*×*M.musculus* F1 mice. The proportion of methylation at each CpG dinucleotide in a WT mouse clearly demonstrates that the level of methylation increases from the 5′ to the 3′ region of the CpG island (D,E). Analysis of the blastocyst DNA (G) showed an almost complete lack of methylation in region A and in region B. Although parental identity of sequenced alleles for blastocyst DNA could not be determined, the sequenced alleles are most likely derived from both parents since no bias of amplification was observed in adult tissues. In sperm DNA (H) and in ovulated oocytes (I), none of the 42 CpG dinucleotides were ever methylated, consistent with the complete absence of methylation in blastocysts and on the adult brain and kidney paternal alleles. The number of analyzed alleles is indicated (n).
(TIF)

Figure S10 Competition between “active” *Ndn* promoters leading to an allelic exclusion model of *Ndn* expression in mouse brain. This scheme summarizes A) the comparison of Ndn maternal expression in +m/−p individuals from the *Ndn^tmStw^* mouse line (in which the *Ndn*-coding part has been replaced by *B-Gal* coding part) versus the *Ndn^tm1.Mus1^* mouse line (in which the *Ndn*-promoter and part of the *Ndn*-coding part have been deleted) and B) the level of eGFP expression from a non-imprinted BAC-*Ndn*-eGFP transgene (integrated on an autosomal chromosome) in 3 mouse lines that present different levels of endogenous expression of *Ndn*: *Ndn* over-expression (*Ndn*++, a), normal expression (WT,b) and no *Ndn* expression (*Ndn−/−*, c). The maternal *Ndn* allele and transcripts are in red, the paternal *Ndn* allele and transcripts are in blue, the *B-Gal* transcripts are in yellow and the *eGFP* transcripts are in green. The maternal promoter is methylated (grey box, CH3), however when the maternal allele is expressed we did not observe a gross modification of this methylation profile in the whole brain. However we cannot exclude a loss of methylation in the Ndn promoter in specific cells leading to an open methylation status (??).
(TIF)

Table S1 Cinicopathological details of Prader-Willi Syndrome and control subjects. BMI, body mass index; M, male; NBB no, Netherlands Brain bank number; ND, not determined; PMD, post-mortem delay; SIDS, Sudden Infant Death Syndrome.
(DOCX)

Table S2 Sequence of the QUASEP primers.
(DOCX)
