# Supplementary figures and images for: Stochastic Loss of Silencing of the Imprinted Ndn/NDN Allele, in a Mouse Model and Humans with Prader-Willi Syndrome, Has Functional Consequences
Source: PLoS Genet. 2013 Sep 5;9(9):e1003752. doi: 10.1371/journal.pgen.1003752 (PMC3764186; doi:10.1371/journal.pgen.1003752)

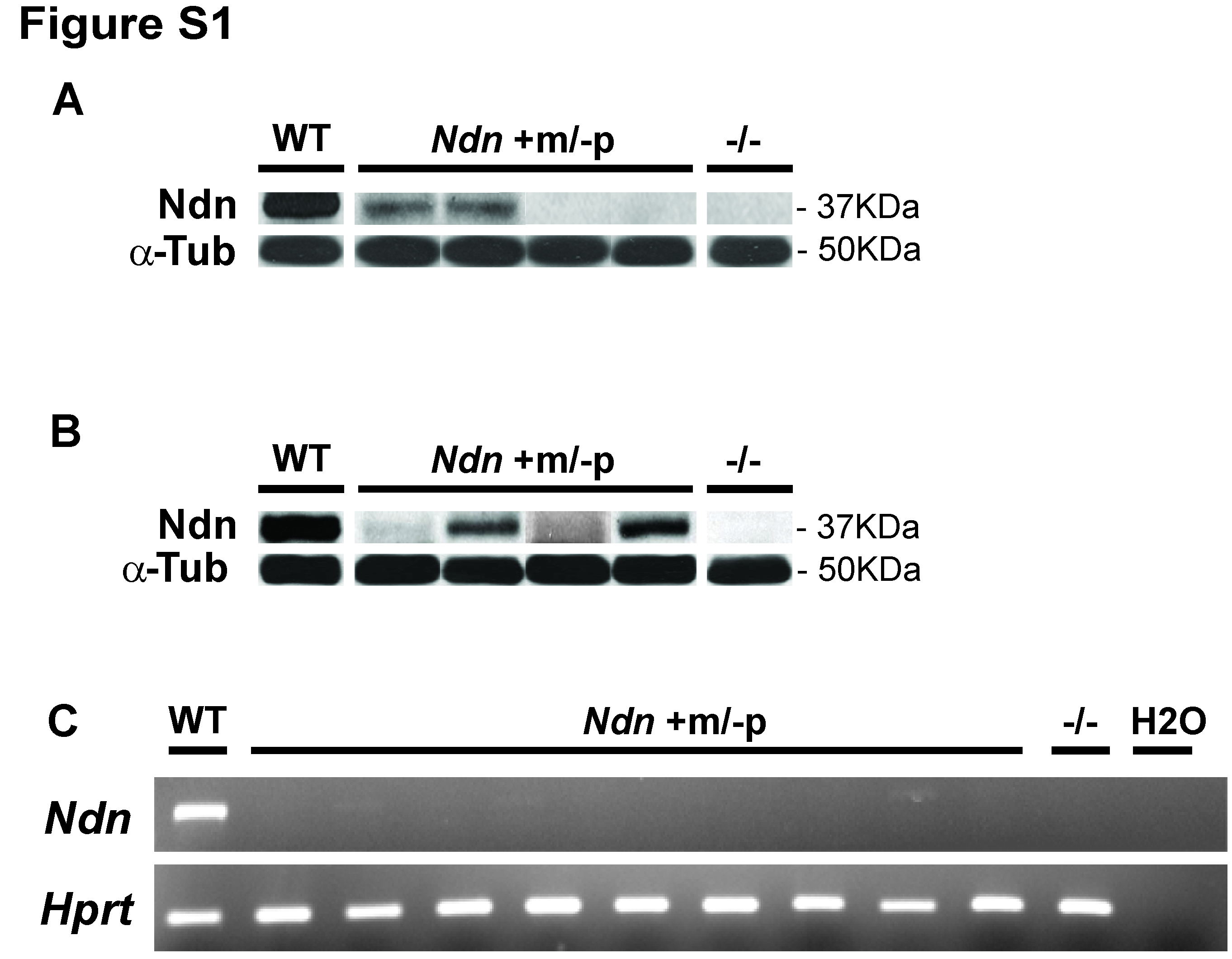

Supplement: Figure S1 — Ndn expression analyzed by RT-PCR and Western blot. (A and B) Western-blot analysis on homogenates from P1 whole-brain (A) or from E12.5 embryos (B) using an anti-Necdin antibody and an anti-Tubulin antibody as positive control. Necdin-specific immunoreactivity (at 37 Kd) was visible in all WT and in four Ndn+m/−p mice, while four Ndn+m/−p and Ndn−/− mutants showed no Necdin expression (A,B). (C) RT-PCR analysis to detect a 562 bp fragment of Ndn transcripts in whole brain of WT, Ndn+m/−p and Ndn−/− neonates. Note the complete absence of Ndn expression from the maternal allele in the neonatal brain using this approach. A positive control PCR was performed using Hprt primers in order to amplify a 429 bp fragment of Hprt transcripts (C, Hprt). (TIF) [file pgen.1003752.s001.tif]

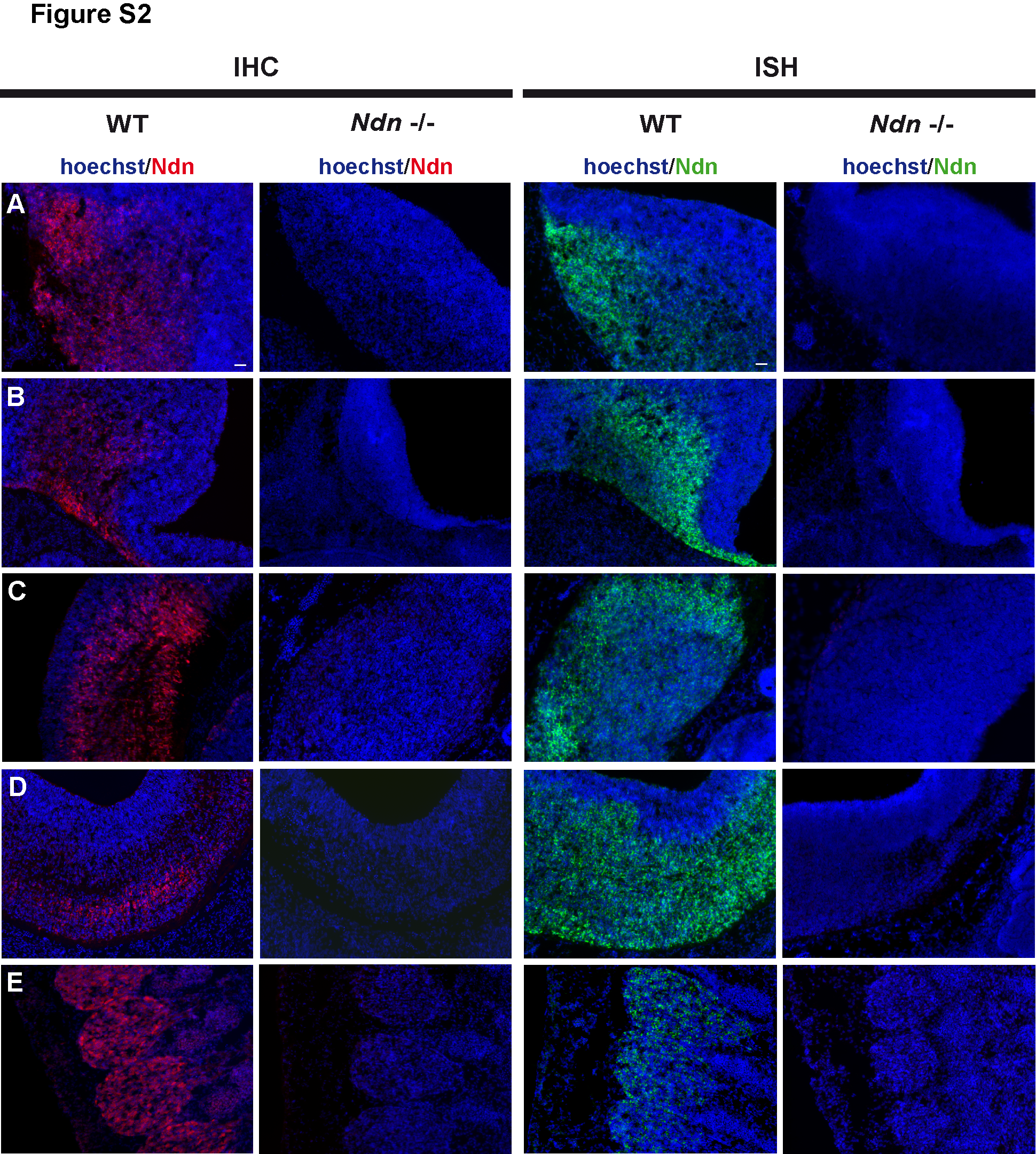

Supplement: Figure S2 — Ndn expression in Ndn−/− E12.5 embryos. Expression of Ndn in the nervous system of WT and Ndn−/− embryos at E12.5 revealed by IHC or ISH on frozen sections using an anti-Necdin antibody (red) or Ndn RNA probe (green). Tissue sections are visualized using a Hoechst labeling (blue). Although expression is detected in WT embryos at the protein and transcript levels in the preoptic area (A), supraoptic area (B), thalamus (C), pons (D) and in the dorsal root ganglia (E), no transcripts or protein are detected in Ndn −/− embryos. Scale bar: 50 µm. (TIF) [file pgen.1003752.s002.tif]

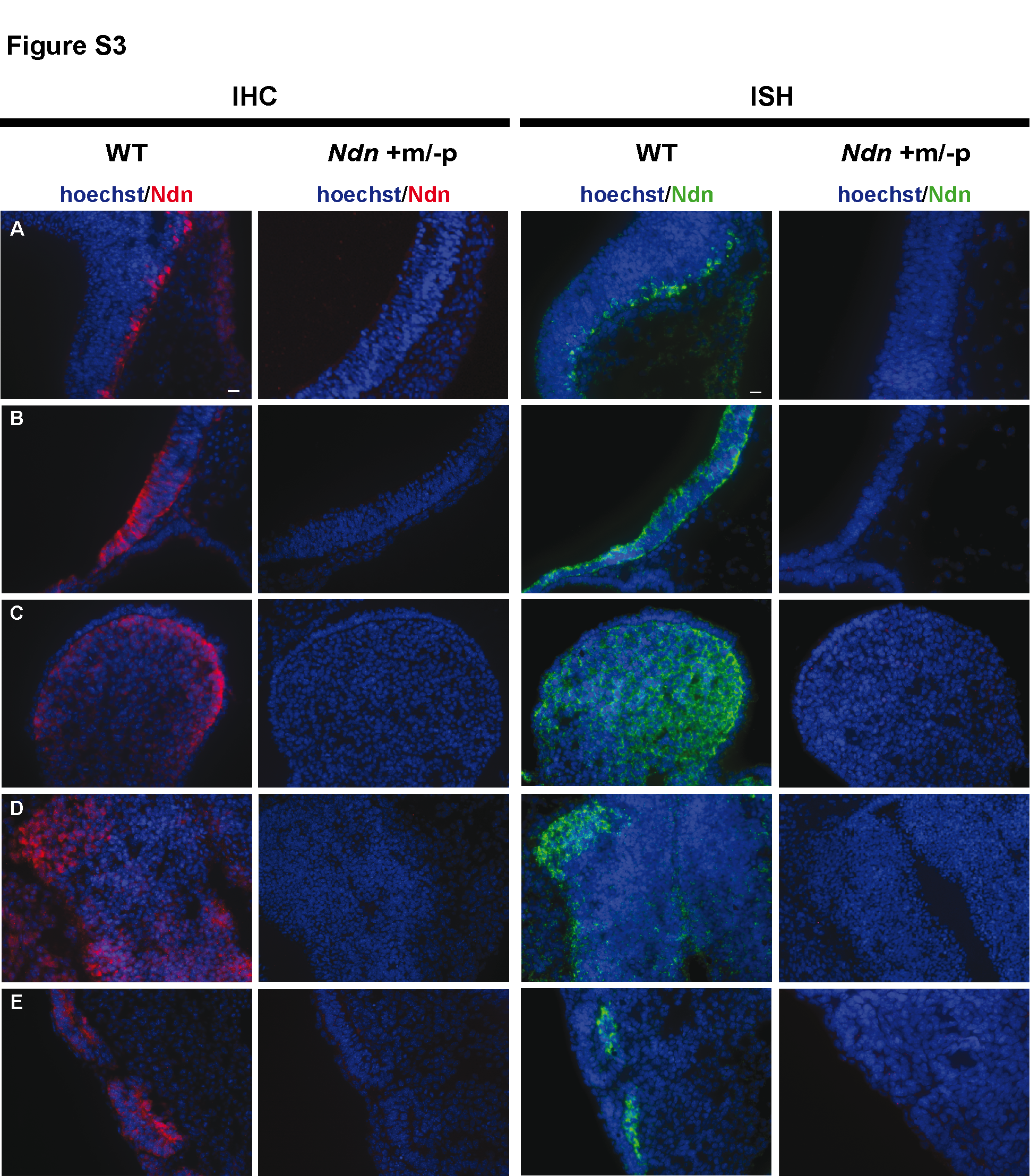

Supplement: Figure S3 — Ndn expression in Ndn+m/−p E10.5 embryos. Expression of Necdin in the nervous system of WT and Ndn+m/−p Ndn embryos at E10.5 revealed by IHC or ISH on frozen sections using an anti-Necdin antibody (red) or a Ndn RNA probe (green). Tissue sections are visualized using a Hoechst labeling (blue). Expression is detected in WT embryos at the protein and transcript levels in the preoptic area (A), supraoptic area (B), thalamus (C), pons (D) and in the dorsal root ganglia (E). No expression is found in Ndn+m/−p embryos (n = 9). Scale bar: 50 µm. (TIF) [file pgen.1003752.s003.tif]

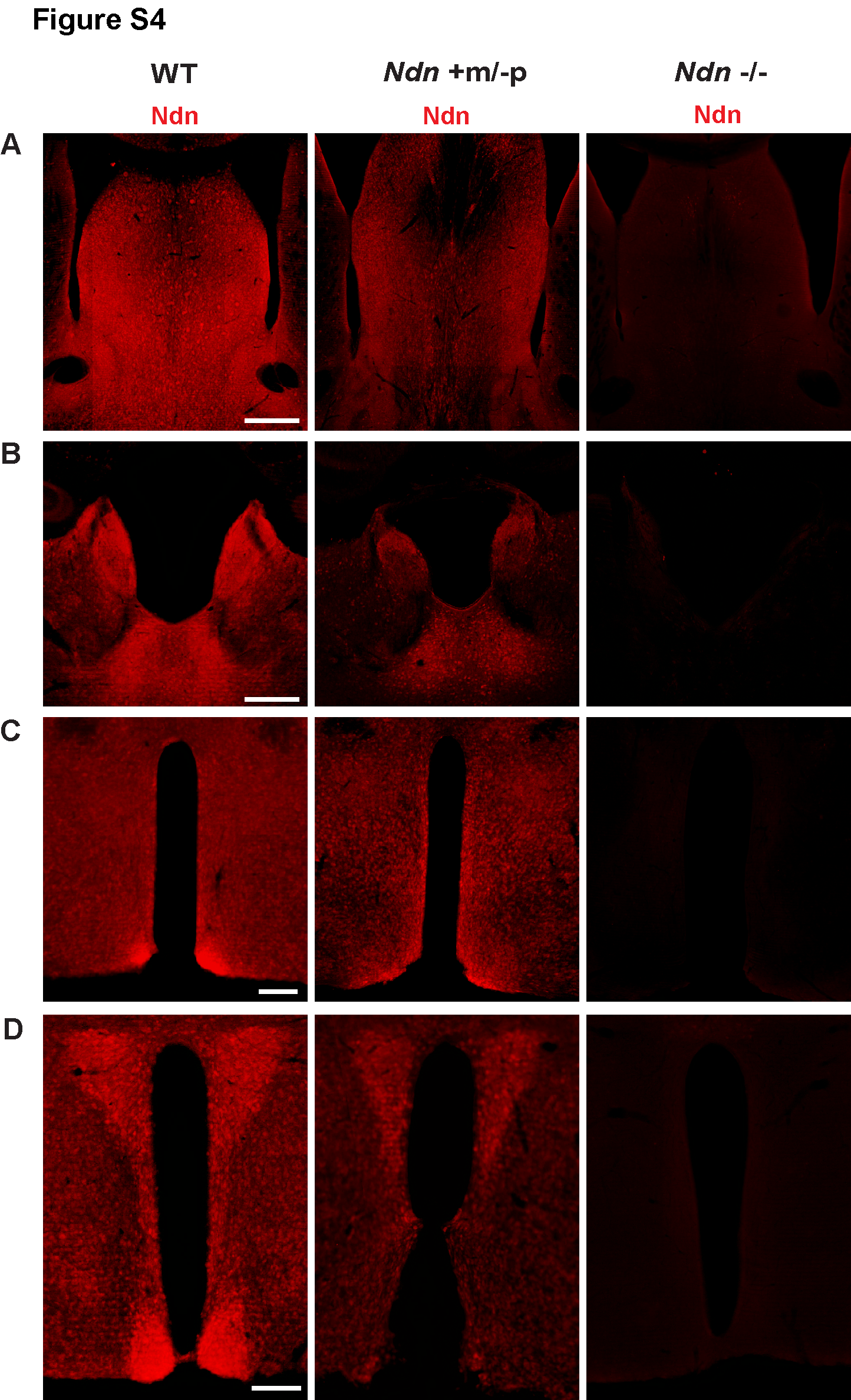

Supplement: Figure S4 — Necdin expression in Ndn+m/−p adult brains. Expression of Necdin in WT, Ndn+m/−p and Ndn−/− adult brains revealed by IHC using an anti-Necdin antibody (in red) on coronal sections at the lateral septum level (A), nucleus of thalamus (B), arcuate nucleus of hypothalamus (C), paraventricular hypothalamic nucleus (D) and suprachiasmatic nucleus (D). Scale bar: 500 µm (A), 250 µm (B), 200 µm (C and D). (TIF) [file pgen.1003752.s004.tif]

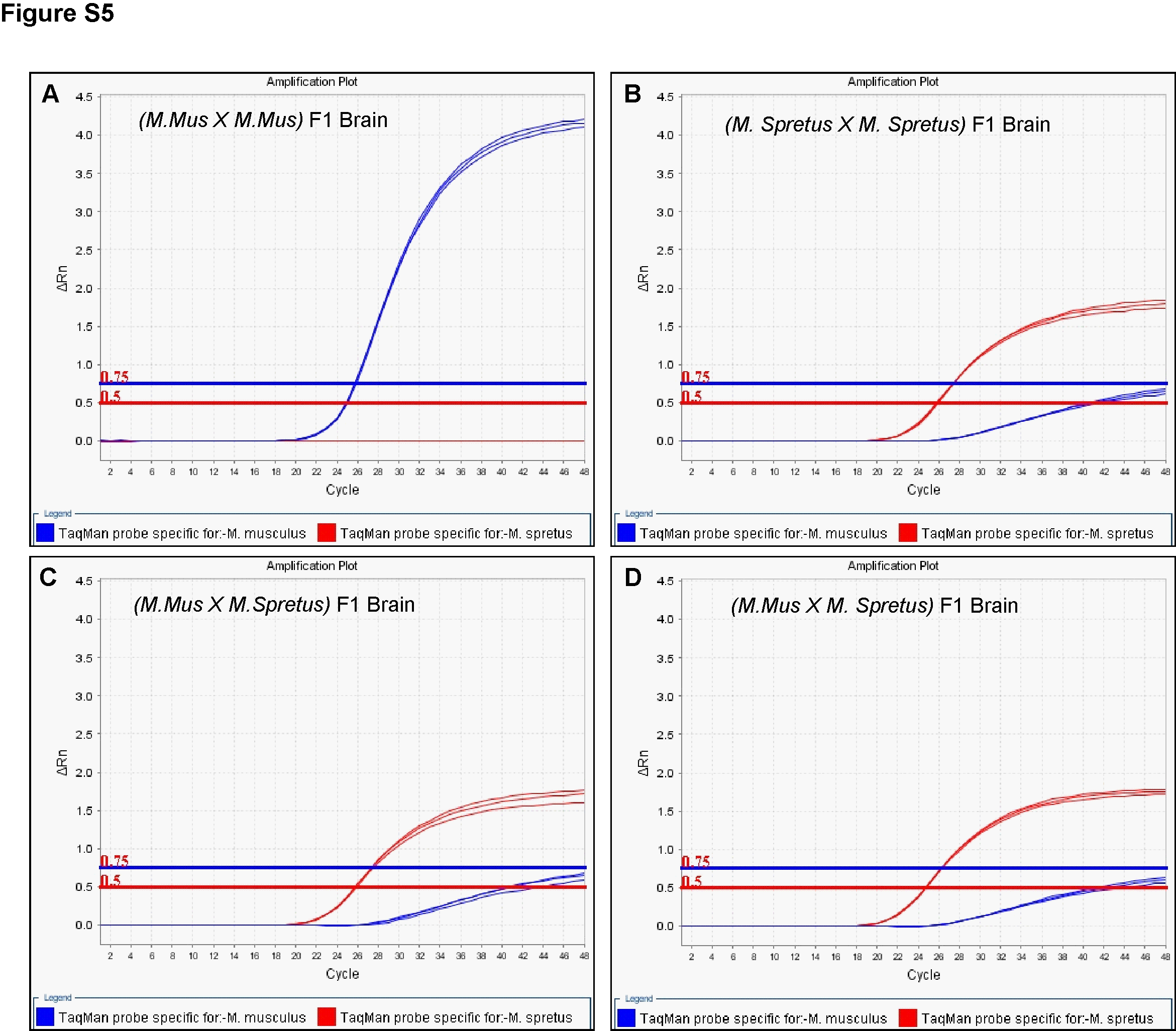

Supplement: Figure S5 — RT-qPCR shows an absence of Ndn maternal allele expression in wild-type mice. TaqMan probe-based RT-qPCR analysis for allele-specific quantification of the 5 bp indel polymorphism on brain cDNA samples of Mus musculus (C57Bl/6J) (A), Mus spretus (B) and two F1 hybrid offspring from a female Mus musculus X male Mus spretus cross (C and D). The blue curve and the horizontal blue line represent transcripts from the Mus musculus allele and the corresponding threshold Ct value, the red curve and the horizontal red line transcripts from the Mus spretus allele and the corresponding threshold Ct value. The results obtained for the brain cDNA samples of the other 30 F1 hybrid mice were identical to those shown in C and D. (TIF) [file pgen.1003752.s005.tif]

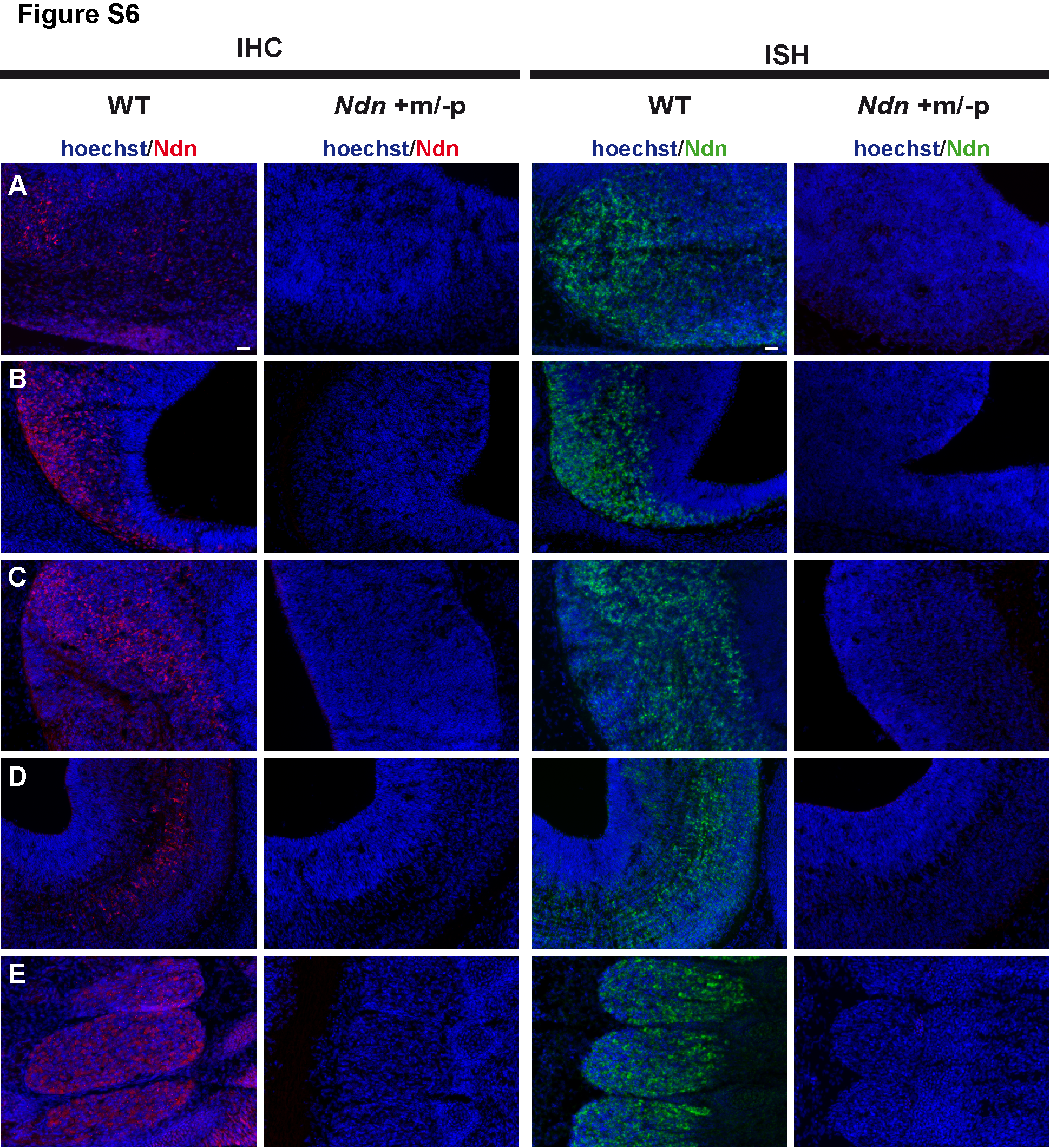

Supplement: Figure S6 — Expression of Ndn in the nervous system of wild-type and Ndn+m/−p Ndntm2Stw embryos at E12.5. We performed IHC or ISH on frozen sections using an anti-Necdin antibody (in red) or Ndn RNA probe (in green). Tissue sections are visualized using a Hoechst labeling (blue). An expression is detected at the protein and transcript levels in WT embryos only, in the septal area (A), preoptic area (B), thalamus (C), pons (D) and in the dorsal root ganglia (E). Note that there is no expression in Ndn+m/−p Ndntm2Stw embryos. Scale bar: 50 µm. (TIF) [file pgen.1003752.s006.tif]

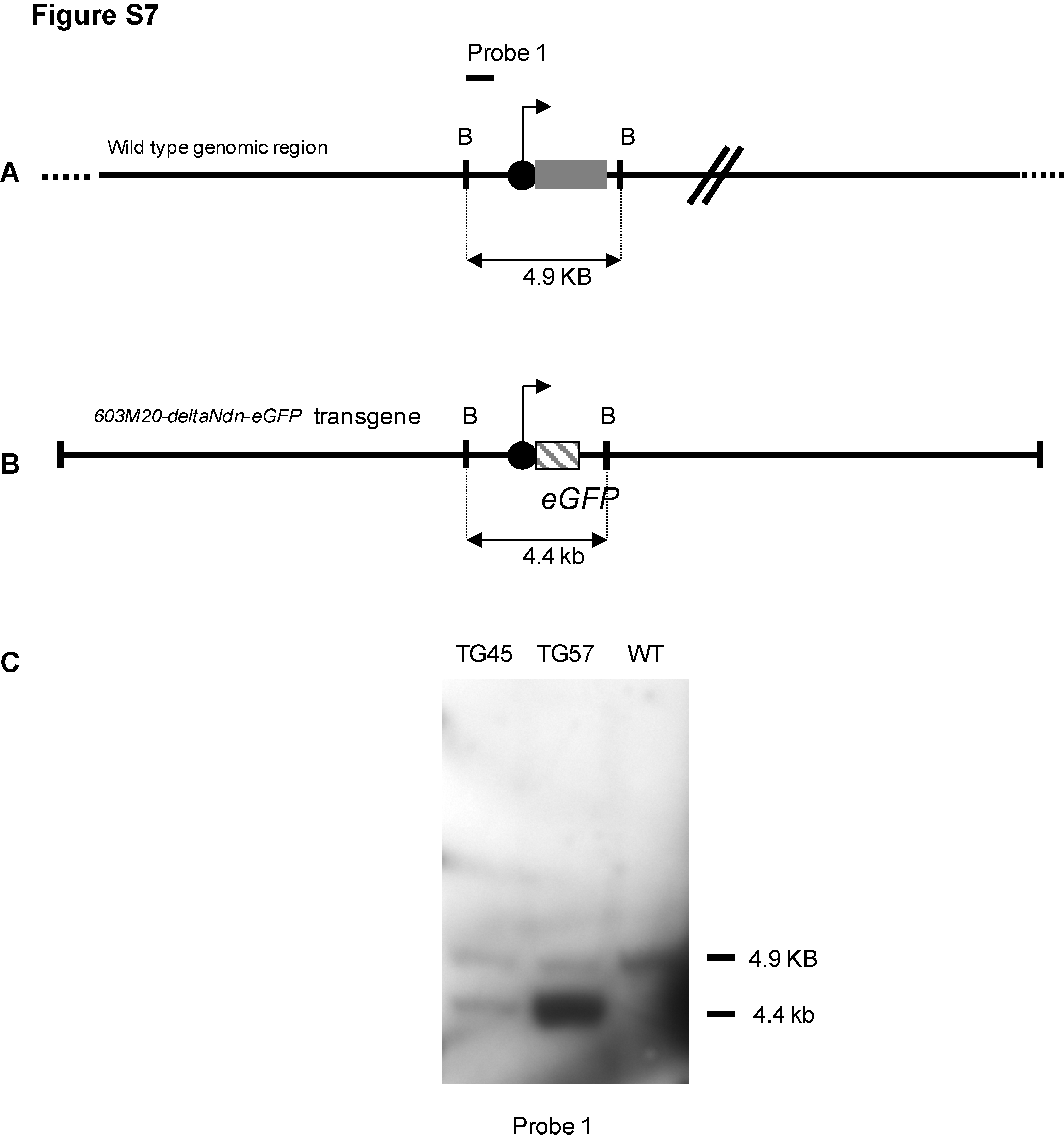

Supplement: Figure S7 — BAC Ndn-eGFP transgene construction and analyses. Structure and copy number of the 603M20-deltaNdn-eGFP transgene in line TG45 (referred in the text to TG) and TG57 (not studied here but shown for comparison to TG45). (A) The Ndn genomic region and (B) 603M20-deltaNdn-eGFP transgene are represented on the upper diagram. (C) Genomic DNA was isolated from WT or transgenic mice (lines 45 and 57), digested by BglII, separated by gel electrophoresis, blotted and hybridized to the probe 1. In line 45, the 4.4 kb transgenic and the 4.9 kb endogenous fragments detected after hybridization were of same intensity, indicating that the transgene was present in 1 or 2 copies although several copies are detected in line 57. Only the TG45 mouse line expresses the eGFP transgene and was used in our experiments. (TIF) [file pgen.1003752.s007.tif]

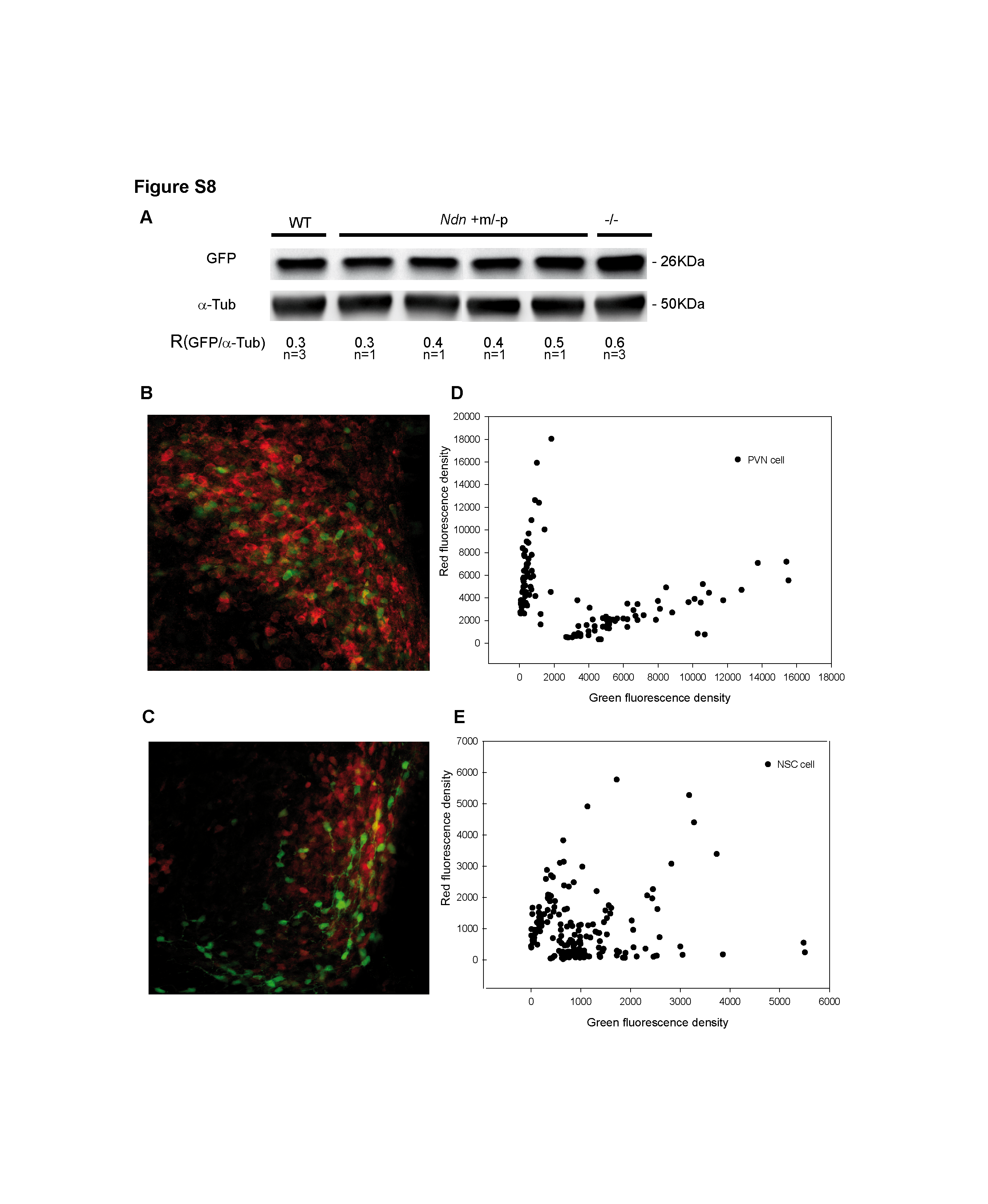

Supplement: Figure S8 — Quantitative analyses of eGFP expression and Necdin expression in the hypothalamus of WT TG+ mice. (A) Western blot analysis to quantify the eGFP expression relative to α-Tubulin in WT, Ndn+m/−p and, Ndn−/− hypothalamus of TG+ mice. The ratio of eGFP/α Tubulin (R) was calculated for each Ndn+m/−p individual and a mean (n = 3) is given for the WT and Ndn−/− genotypes. R is an indicator of the hypothalamic quantity of eGFP per genotype. (B,C) Immunofluorescence (green) of the Ndn-eGFP transgene and immunolabeling of the Necdin-positive cells (red) in the PVN (b) or the NSC (C). (D,E) Graph of the quantification of green and red fluorescence performed for each individual cell from the PVN (D) and NSC (E) brain structures. (TIF) [file pgen.1003752.s008.tif]

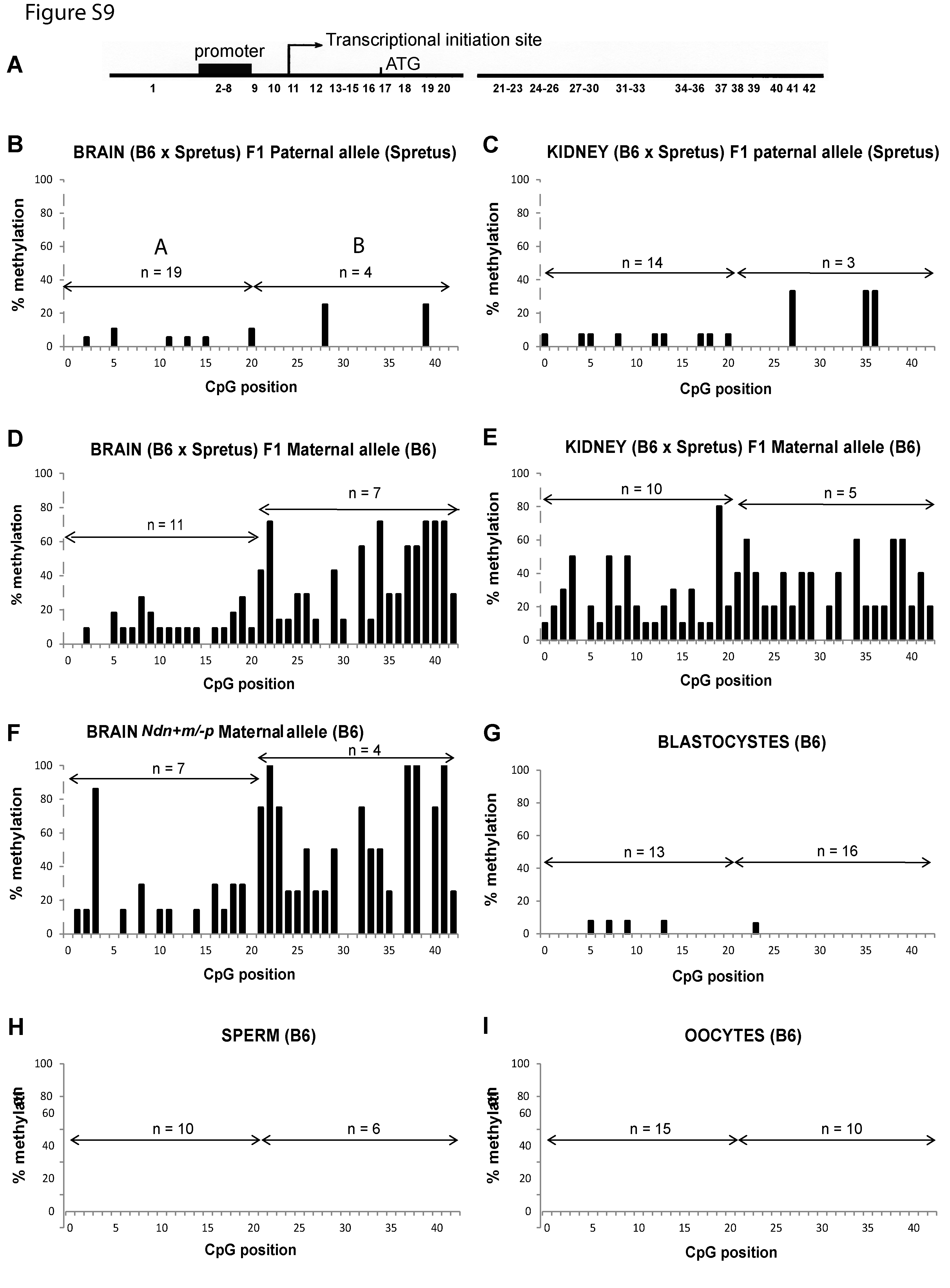

Supplement: Figure S9 — Methylation profile at the Ndn DMR. (A) Localization of CpG dinucleotides in relation to the Ndn transcriptional start site, translational start site and promoter is represented. The transcriptional start site is between CpG sites 10 and 11, and the translational start site (ATG) between CpG sites 16 and 17. (B) and (C) CpG dinucleotides are very sparsely methylated or completely unmethylated in regions A and B of the paternal alleles in brain (B) and kidney (C). In regions A and B, the maternal alleles display a much higher level of CpG methylation, the average percentage of methylated CpG being 44% in region B in brain (D) and kidney (E) of WT mice as in brain of Ndn+m/−p mice (F). It should be noted that no particular bias of amplification in either region A or B of paternal versus maternal alleles was observed and that the maternal methylation profiles described were identical in interspecific M. spretus×M.musculus F1 mice. The proportion of methylation at each CpG dinucleotide in a WT mouse clearly demonstrates that the level of methylation increases from the 5′ to the 3′ region of the CpG island (D,E). Analysis of the blastocyst DNA (G) showed an almost complete lack of methylation in region A and in region B. Although parental identity of sequenced alleles for blastocyst DNA could not be determined, the sequenced alleles are most likely derived from both parents since no bias of amplification was observed in adult tissues. In sperm DNA (H) and in ovulated oocytes (I), none of the 42 CpG dinucleotides were ever methylated, consistent with the complete absence of methylation in blastocysts and on the adult brain and kidney paternal alleles. The number of analyzed alleles is indicated (n). (TIF) [file pgen.1003752.s009.tif]

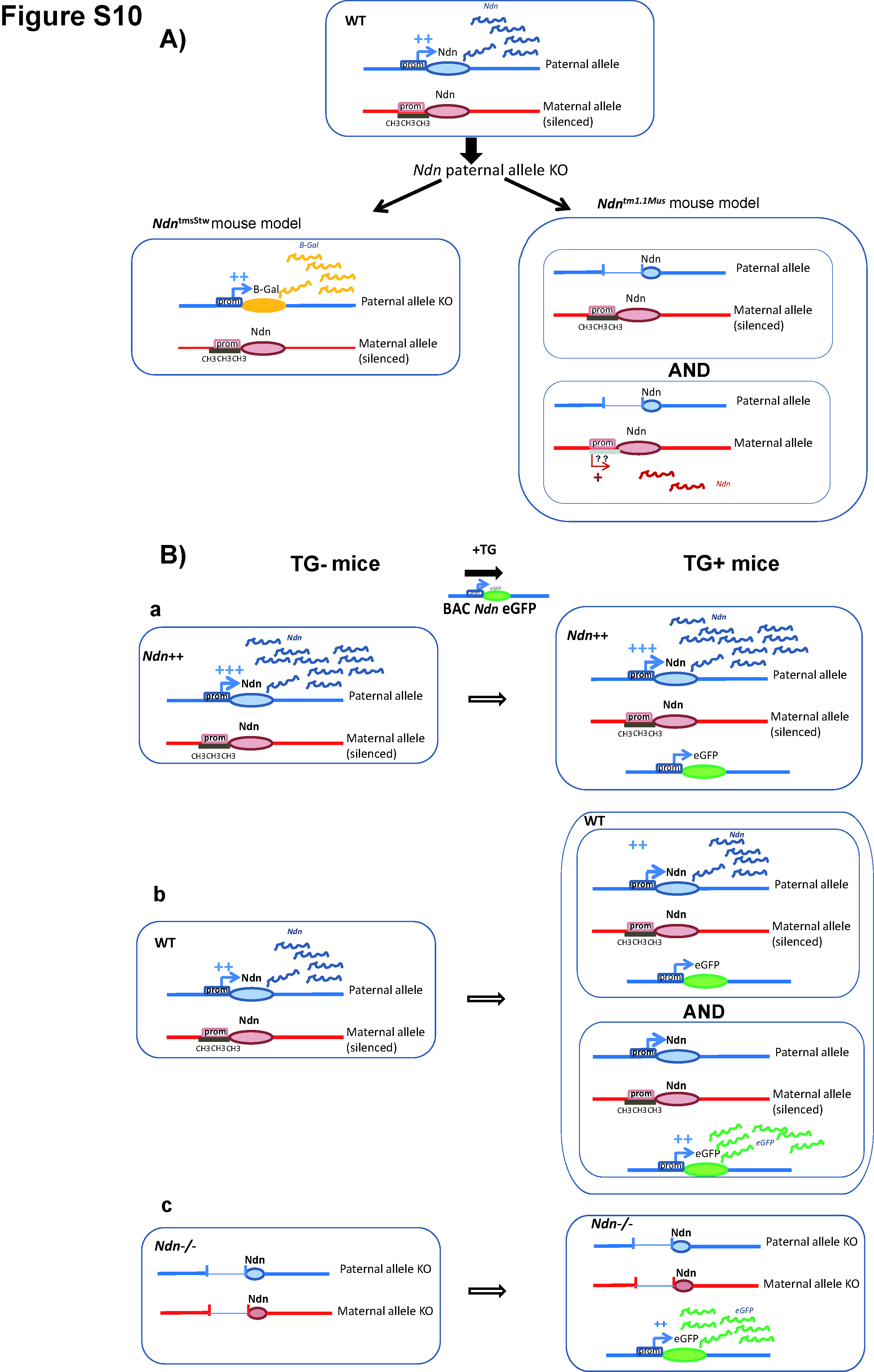

Supplement: Figure S10 — Competition between “active” Ndn promoters leading to an allelic exclusion model of Ndn expression in mouse brain. This scheme summarizes A) the comparison of Ndn maternal expression in +m/−p individuals from the NdntmStw mouse line (in which the Ndn-coding part has been replaced by B-Gal coding part) versus the Ndntm1.Mus1 mouse line (in which the Ndn-promoter and part of the Ndn-coding part have been deleted) and B) the level of eGFP expression from a non-imprinted BAC-Ndn-eGFP transgene (integrated on an autosomal chromosome) in 3 mouse lines that present different levels of endogenous expression of Ndn: Ndn over-expression (Ndn++, a), normal expression (WT,b) and no Ndn expression (Ndn−/−, c). The maternal Ndn allele and transcripts are in red, the paternal Ndn allele and transcripts are in blue, the B-Gal transcripts are in yellow and the eGFP transcripts are in green. The maternal promoter is methylated (grey box, CH3), however when the maternal allele is expressed we did not observe a gross modification of this methylation profile in the whole brain. However we cannot exclude a loss of methylation in the Ndn promoter in specific cells leading to an open methylation status (??). (TIF) [file pgen.1003752.s010.tif]
