# Supplementary material for: Stochastic Loss of Silencing of the Imprinted Ndn/NDN Allele, in a Mouse Model and Humans with Prader-Willi Syndrome, Has Functional Consequences
Source: PLoS Genet. 2013 Sep 5;9(9):e1003752. doi: 10.1371/journal.pgen.1003752 (PMC3764186; doi:10.1371/journal.pgen.1003752)
Supplement: Table S1 — Cinicopathological details of Prader-Willi Syndrome and control subjects. BMI, body mass index; M, male; NBB no, Netherlands Brain bank number; ND, not determined; PMD, post-mortem delay; SIDS, Sudden Infant Death Syndrome. (DOCX) [file pgen.1003752.s011.docx]

| **NBB no** | **Sex** | **Age (yr)** | **PMD (hours)** | **Fixation time (days)** | **Brain weight (g)** | **Cause of death** | **Other clinical problem** | **PWS diagnostic test** |
| --- | --- | --- | --- | --- | --- | --- | --- | --- |
| **PWS** | | | | | | | |  |
| 00-028 | M | 32 | <48.0 | 59 | 1550 | Sudden death after 2 days of fever, diarrhea and vomiting | Weight 76 kg, ch 15q11-13 deletion | Lack of parental allele at GABA, GABRA5, D15S97 |
| 95-104 | M | 51 | 16.0 | 32 | 1570 | Pneumonia | Hypertension, testicular seminoma 28 yr, BMI 33.8, ch15 UPD | Ch15UPD, PW71B maternal methylation pattern, FISH: 2 signals on the long arms of both ch15 homologues |
| 99-079 | M | 0.75 | 10.0 | 76 | ND | Pneumonic infiltration | Influenza 1.4 months ago, hypoglycaemia, hypothermia | ch15q11-13 deletion, |
| **Controls** | | | | | |  | |  |
| 88-017 | M | 31 | 96.0 | 27 | 1550 |  |  | No PWS mutation |
| 94118 | M | 49 | 22.3 | 33 | 1254 | Faecal peritonitis post revision ileocolonic anastomosis | Hemicolectomy for metastatic colon adenocarcinoma | No PWS mutation |
| 97-153 | F | 0.6 | 20.7 |  |  | SIDS |  | No PWS mutation |

Table S1
