# Supplementary material for: Stochastic Loss of Silencing of the Imprinted Ndn/NDN Allele, in a Mouse Model and Humans with Prader-Willi Syndrome, Has Functional Consequences
Source: PLoS Genet. 2013 Sep 5;9(9):e1003752. doi: 10.1371/journal.pgen.1003752 (PMC3764186; doi:10.1371/journal.pgen.1003752)
Supplement: Table S2 — Sequence of the QUASEP primers. (DOCX) [file pgen.1003752.s012.docx]

| **QUASEP Primers** | | | |
| --- | --- | --- | --- |
| Assay | forward primer 5'-3' | reverse primer 5'-3' | sequencing primer 5'-3' |
| *Ndn* 3‘-UTR SNP1 *Mus musculus*: G *Mus spretus*: A | Bio -CAGGGGCACACTGATAGTTTCT | CGAAAGCACAAAAGTGAAAGTACC | GCACTGTGGATTTGG |
| *Ndn* 3‘-UTR SNP2 *Mus musculus*: C *Mus spretus*: T | Bio-TTGTTCTTTGTATGGGACTGATG A | TGCTGTTAAGTCCTGCTAACTCTC | CTCCAGGGCCTTCTT |

Table S2
